# A new method based on surface‐sample pollen data for reconstructing palaeovegetation patterns

**DOI:** 10.1111/jbi.14448

**Published:** 2022-06-15

**Authors:** Esmeralda Cruz‐Silva, Sandy P. Harrison, Elena Marinova, I. Colin Prentice

**Affiliations:** ^1^ School of Archaeology, Geography & Environmental Science Reading University Reading UK; ^2^ Laboratory for Archaeobotany Baden‐Württemberg Cultural Heritage State Office Geienhofen‐Hemmenhofen Germany; ^3^ Georgina Mace Centre for the Living Planet, Department of Life Sciences Imperial College London Ascot UK

**Keywords:** biomes, biomisation, Eastern Mediterranean, EMBSeCBIO, palaeovegetation, pollen data, potential natural vegetation, vegetation change, vegetation reconstruction

## Abstract

**Aim:**

Biomisation has been the most widely used technique to reconstruct past regional vegetation patterns because it does not require an extensive modern pollen dataset. However, it has well‐known limitations including its dependence on expert judgement for the assignment of pollen taxa to plant functional types (PFTs) and PFTs to biomes. Here we present a new method that combines the strengths of biomisation with those of the alternative dissimilarity‐based techniques.

**Location:**

The Eastern Mediterranean‐Black Sea Caspian Corridor (EMBSeCBIO).

**Taxon:**

Plants

**Methods:**

Modern pollen samples, assigned to biomes based on potential natural vegetation data, are used to characterize the within‐biome means and standard deviations of the abundances of each taxon. These values are used to calculate a dissimilarity index between any pollen sample and every biome, and thus assign the sample to the most likely biome. We calculate a threshold value for each modern biome; fossil samples with scores below the threshold for all modern biomes are thus identified as non‐analogue vegetation. We applied the new method to the EMBSeCBIO region to compare its performance with existing reconstructions.

**Results:**

The method captured changes in the importance of individual taxa along environmental gradients. The balanced accuracy obtained for the EMBSeCBIO region using the new method was better than obtained using biomisation (77% vs. 65%). When the method was applied to high‐resolution fossil records, 70% of the entities showed more temporally stable biome assignments than obtained using biomisation. The technique also identified likely non‐analogue assemblages in a synthetic modern dataset and in fossil records.

**Main conclusions:**

The new method yields more accurate and stable reconstructions of vegetation than biomisation. It requires an extensive modern pollen dataset, but is conceptually simple, and avoids subjective choices about taxon allocations to PFTs and PFTs to biomes.

## 1 INTRODUCTION

Pollen evidence has been widely used to reconstruct Holocene changes in vegetation, both at individual sites and at a regional scale (e.g. Bozilova & Tonkov, 1995; Edwards et al., 2017; Huntley & Birks, 1983). These reconstructions provide insights into how vegetation responds to climate changes and human activities (Gaillard et al., 2010; Prentice, 1986), relevant for understanding how vegetation patterns and biodiversity may respond to future climate changes (Bradshaw et al., 2015; Cole, 2010). They also provide information about the resource base for human societies, relevant for understanding how cultural changes such as the adoption of agriculture affected the natural environment, and documenting the sensitivity of human societies to environmental change and degradation (Leroy et al., 2010; Turner & Sabloff, 2012). Vegetation reconstructions have also been widely used to test the performance of Earth System models (Foley et al., 2013; Kaplan et al., 2003; Song et al., 2021).

Available methods use modern pollen–vegetation relationships to reconstruct regional vegetation changes. Semi‐quantitative approaches, including biomisation (Prentice et al., 1996, 2000), distinguish between vegetation types by grouping individual taxa into plant functional types (PFTs) and PFTs into biomes or Land Cover Classes (LCC) (pseudobiomisation: Fyfe et al., 2010). Quantitative approaches, based on pollen source area theory (Prentice, 1985; Prentice & Parsons, 1983; Sugita, 1993), include the Landscape Reconstruction Algorithms (Sugita, 2007a, 2007b) that accounts for pollen dispersal dynamics using region‐specific pollen productivity estimates (PPEs) and pollen size and deposition velocities, or the Regional Estimates of VEgetation Abundance from Large Sites (REVEALS) without PPEs method (ROPES: Theuerkauf & Couwenberg, 2018), which derives PPEs and mean plant abundances from single pollen records. Lack of information on PPEs and deposition velocities means these model‐based reconstructions have only been applied in limited regions and for limited vegetation classes (Gaillard et al., 2010; Harrison et al., 2020). Statistical approaches including the Modern Analogue Technique (MAT; Gaillard et al., 1994; Jackson & Williams, 2004; Overpeck et al., 1985, 1992; Wang et al., 2020; Williams & Jackson, 2007) use modern training datasets to establish relationships between pollen assemblages and biomes, and then use these relationships to reconstruct biomes for fossil pollen assemblages. The accuracy of statistical reconstructions depends on the representativeness of the training dataset (Turner et al., 2021); the lack of sufficiently extensive modern training data precludes MAT reconstructions for many regions. Biomisation has been one of the most widely used vegetation‐reconstruction methods (Allen & Huntley, 2009; Allen et al., 2000; Bigelow et al., 2003; Edwards et al., 2000; Elenga et al., 2000; Harrison et al., 2001; Jolly et al., 1998; Marchant et al., 2001, 2009; Pickett et al., 2004; Prentice & Webb, 1998; Prentice et al., 1996; Takahara et al., 2000; Tarasov et al., 1998, 2013; Thompson & Anderson, 2000; Williams et al., 1998, 2000; Yu et al., 1998, 2000), in large part because of its conceptual simplicity and minimal data requirements. Although modern pollen samples are used to test the method, biomisation does not require the extensive modern calibration datasets needed for statistical approaches, nor does it require regional information about pollen productivity and deposition velocities.

The principle of the biomisation method is to assign pollen taxa to PFTs defined based on life form, leaf type, phenology and climate tolerance, where taxa within a PFT are assumed to have similar responses to physical and biotic environmental factors. Major vegetation types (biomes) are then characterized as assemblages of PFTs, where the PFTs included represent either the dominant taxa or taxa that are characteristic of the biome. The allocation of pollen taxa into PFTs and PFTs into biomes is initially determined by knowledge of the regional vegetation and subsequently optimized, after comparison of reconstructions to observed vegetation, by removing non‐diagnostic taxa or PFTs. The assignment of individual pollen samples to biomes is made by calculating an affinity score for the similarity of the pollen assemblage to every biome, based on the weighted average of the square root of the pollen abundances of taxa that could be present in each biome.

Biomisation has been shown to produce robust reconstructions of vegetation patterns at key times in the past. However, it has some well‐known limitations. These include the fact that there is no direct relationship between pollen abundance and plant abundance such that the dominant taxa in the regional vegetation may be under‐represented in the pollen assemblages. For example, *Larix* is the dominant genus in the boreal cold‐deciduous forest of Eurasia but produces little pollen and is systematically under‐represented in pollen samples (Bigelow et al., 2003). In contrast, *Pinus* produces abundant and easily dispersed pollen and is often over‐represented in pollen samples, both in biomes where it actually occurs and as a result of long‐distance transport into biomes representing open vegetation types (Bigelow et al., 2003; Edwards et al., 2000). The use of the square root of pollen abundance in the biomisation formula decreases the weight of over‐represented taxa, but they still contribute to biomes that contain the PFT to which they belong. An alternative method of down‐weighting over‐represented taxa is to set a universal threshold of abundance for inclusion in the analysis higher than the 0.5% default value (Williams et al., 2000), but this eliminates a large number of under‐represented taxa that might be diagnostic.

The definition of biomes in terms of diagnostic or dominant PFTs emphasizes the most characteristic expression of a biome but does not account for the fact that there is variability in the abundance of these PFTs within a biome and considerable differences between PFT abundance at, for example, the northern and southern limits of the biome (Edwards et al., 2000). This issue has been solved by creating additional PFTs or biomes. For example, the temperate summergreen PFT in the eastern United States and Canada was split into intermediate and warm variants to separate out taxa with different climatic tolerances (Williams et al., 2000). Similarly, the tundra (TUND) biome has been split into multiple sub‐biomes which are differentiated by the presence/absence of different categories of shrubs (Bigelow et al., 2003). However, these approaches still only consider part of the within‐biome variability.

A further problem is associated with the fact that some biomes are characterized by a subset of the PFTs present in another biome. For example, the PFTs defining deciduous forest types are often a subset of those defining equivalent mixed forest types, creating a situation where identical affinity scores are obtained for the two biomes. The biomisation approach solves this through a tie‐break rule that favours the less PFT‐rich biome. However, the presence of a small amount of pollen from a PFT that is not shared would be sufficient to change the affinity score and hence the biome allocation, meaning that the biome assignments can be sensitive to small changes in pollen abundance and may not be stable. Small changes in pollen abundance can also result in shifts towards biomes that are not characterized by a subset of the PFTs representative of another biome, for example in the case where taxa are assigned to more than one PFT. The problem, referred to as the ‘flickering switch’ problem, is most noticeable when biomisation is used to reconstruct changes in vegetation through time using down‐core pollen samples at a single site (Allen et al., 2000; Fyfe et al., 2018). The flickering switch problem has been observed to be acute in high‐resolution records where one might expect greater similarity in biome allocation between adjacent samples (see e.g. Allen & Huntley, 2009; Marchant et al., 2001).

One final issue poorly addressed by the biomisation technique is the existence of palaeovegetation communities with no analogue in the present‐day vegetation because they consist of extant species but in combinations not found at present (Jackson & Overpeck, 2000; Overpeck et al., 1992; Williams & Jackson, 2007). Statistical dissimilarity approaches have addressed this by establishing thresholds for detecting such assemblages, but it is important then to establish criteria to avoid ad hoc choices of such thresholds (Gavin et al., 2003).

In this study, we have derived a method that overcomes these problems and extends existing dissimilarity‐based approaches by accounting explicitly for within‐biome variability. We calculated a dissimilarity index (which takes account of this variability) between a given pollen sample and every biome, and thereby assessed the likelihood that a sample belongs to a particular biome. We applied this method to reconstruct the modern vegetation of the Eastern Mediterranean‐Black Sea Caspian Corridor (EMBSeCBIO) region (33°–49°N, 20°–60°E), where the performance of the new approach can be compared to reconstructions made using biomisation (Marinova et al., 2018). The EMBSeCBIO region is characterized by strong temperature and precipitation gradients and topographic heterogeneity, resulting in clear patterns in biome distribution within a relatively limited geographical space and thus provides an excellent case to test how well the different methods capture spatially complex vegetation patterns.

## 2 MATERIALS AND METHODS

**FIGURE 1:**
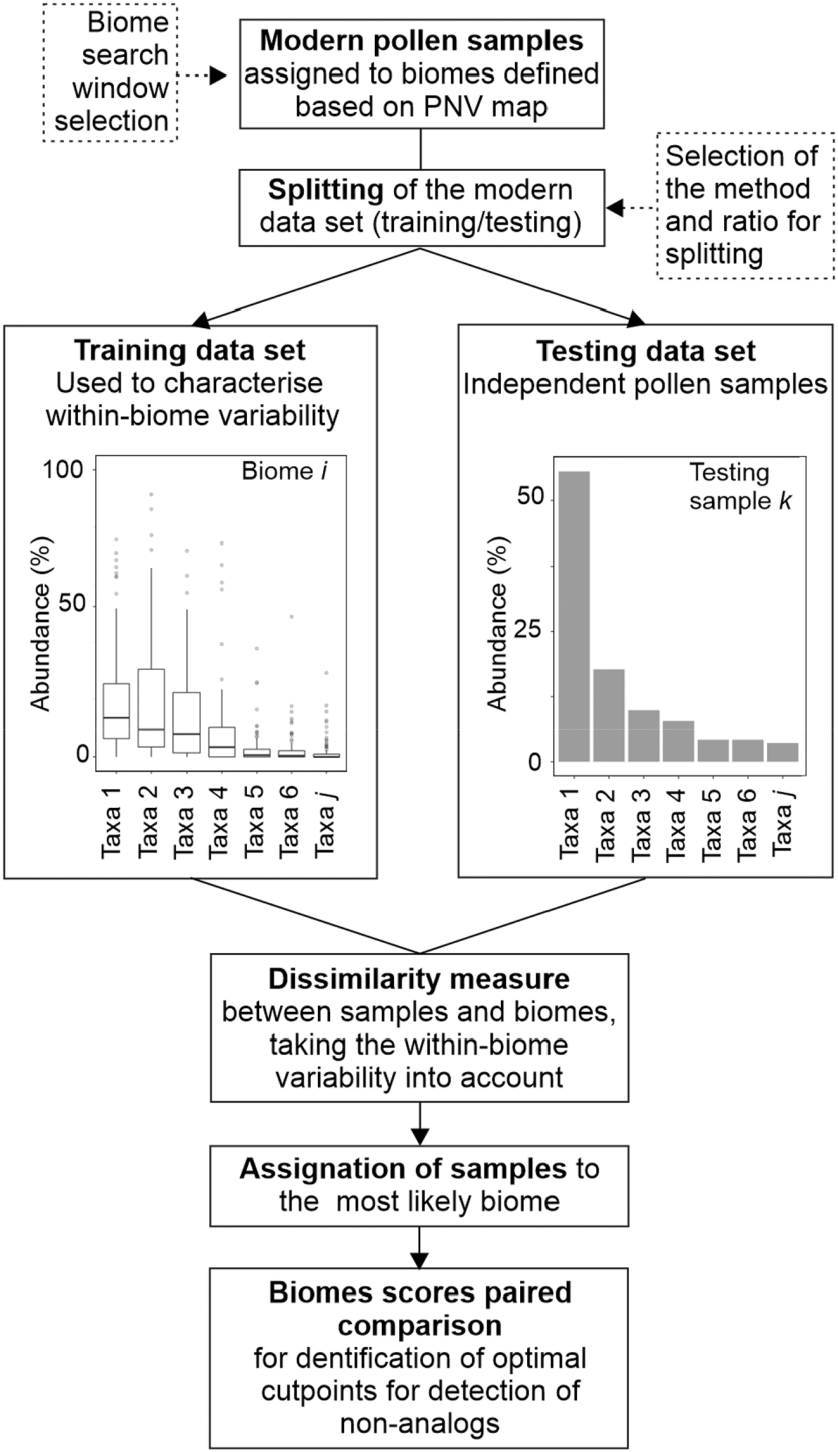
Overview of the approach for pollen‐based vegetation reconstruction accounting for within‐biome variability. PNV, potential natural vegetation.

The workflow is summarized in Figure 1. We first assigned modern pollen samples to biomes, where the biomes are defined using a recently developed global modern potential vegetation map. Since the modern samples were derived from different settings and basins of different sizes, we tested what would be an appropriate search window for determining the biome for each sample. We then divided the modern pollen samples into training and testing datasets. The training dataset was used to characterize the within‐biome variability of the pollen assemblages assigned to a given biome, in terms of the mean, range and standard deviation of each pollen taxon. We then calculated the dissimilarity index between every sample in the test dataset and every biome. This index provides an approximation of the probability that a given pollen assemblage would be produced by a particular biome such that samples can be allocated to the biome with the highest likelihood. We tested the robustness of these assignments using different training and testing data, since the selection and size of the training and testing datasets could influence the within‐biome variability and hence the dissimilarity calculation. We then compared the reconstructions based on this new approach to previous vegetation reconstructions for the EMBSeCBIO region. Finally, we estimated optimal threshold values for the biome scores by pairwise comparison of every biome, using the receiver operating characteristic (ROC) curve as a performance metric, to determine whether assemblages were characteristic of an observed modern biome or could represent a non‐analogue vegetation type. We tested this procedure using a synthetic modern dataset and also applied it to fossil pollen assemblages from the EMBSeCBIO region.

### 2.1 Pollen and vegetation data

**FIGURE 2:**
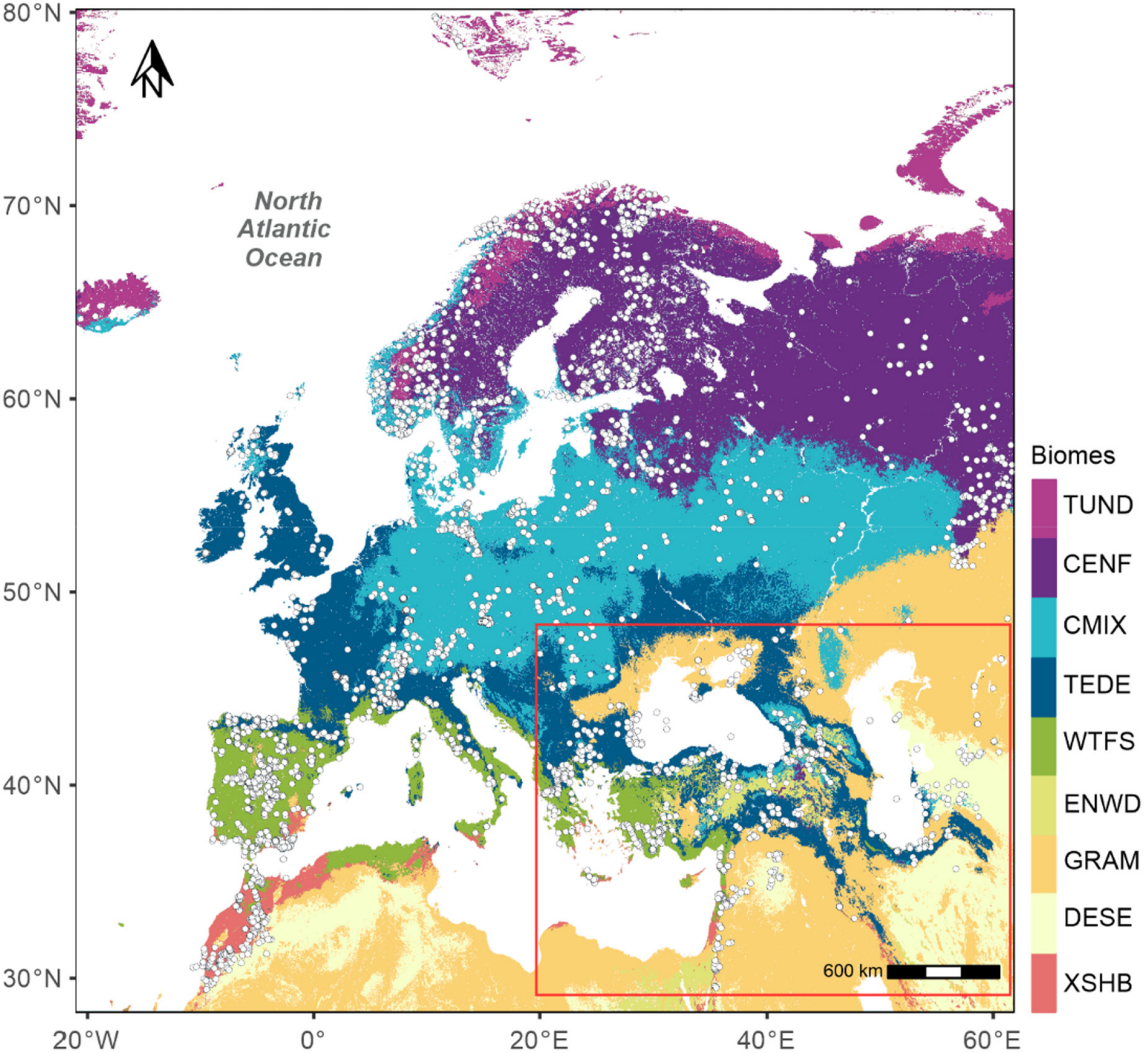
Map of the distribution of modern pollen samples from the SPECIAL modern pollen dataset (SMPDS) and the eastern Mediterranean‐Black Sea Caspian corridor (EMBSeCBIO) database used for the training and testing datasets. The red box delineates the area covered by the EMBSeCBIO database; samples outside the red box were obtained from the SMPDS dataset. The background map shows the distribution of major biomes derived from the Hengl et al. (2018) reconstruction of potential natural vegetation. The biome codes are: CENF, cold evergreen needleleaf forest; CMIX, cool mixed evergreen needleleaf and deciduous broadleaf forest; DESE, desert; ENWD, evergreen needleleaf woodland; GRAM, graminoids with forbs; TEDE, temperate deciduous malacophyll broadleaf forest; TUND, tundra; WTFS, warm‐temperate evergreen needleleaf and sclerophyll broadleaf forest; XSHB, xeric shrubland.

The modern pollen dataset was derived from the SPECIAL Modern Pollen Data Set (SMPDS: Harrison, 2019) and the EMBSeCBIO pollen database (Harrison & Marinova, 2017; Harrison et al., 2021). The SMPDS consists of relative abundance records of 247 pollen taxa from 6459 terrestrial sites from Europe, the Middle East and northern Eurasia. These taxa result from taxonomic harmonization of the individual records and amalgamation of rare taxa into higher taxonomic groups, after ensuring that this was consistent with their distribution in climate space (Harrison, 2020; Wei et al., 2020). To avoid duplication, we removed all SMPDS samples from the EMBSeCBIO region (28° to 49°N, 20° to 62°E). We did not use SMPDS sites from east of 62°E, where the sampling is limited and likely not representative of the diversity of the vegetation. Most of the pollen assemblages in the SMPDS (94%) are from lake or bog environments where sediments are currently accumulating, have been dated to the last 50 years, or are modern samples from moss polsters, litter or pollen traps. For comparability, we only used sites from the EMBSeCBIO database with ages <150 calibrated years before present (cal BP). This resulted in the selection of 1356 samples from the EMBSeCBIO database and 4409 samples from the SMPDS (Figure 2). Pollen counts in the EMBSeCBIO database were amalgamated into higher taxon groupings consistent with the SMPDS (Table S1).

Since our goal was to develop a method to reconstruct vegetation changes through time, we used potential natural vegetation (PNV) as a target for the modern reconstructions. Maps of PNV have been widely used in this way to test regional vegetation reconstructions (e.g. Bigelow et al., 2003; Marchant et al., 2009; Marinova et al., 2018). The modern vegetation data were extracted from an updated version of the Global PNV map produced by Hengl et al. (2018). The original version of this map had a resolution of 1 km; the updated version (https://github.com/Envirometrix/PNVmaps) has a resolution of 250 m. The PNV map was produced using pollen‐based vegetation reconstructions as a target, a large set of spatially explicit covariate datasets representing the potential climatic, topographic, geologic and hydrological controls on plant growth, and an ensemble of five machine‐learning approaches (neural networks, random forest, gradient boosting, K‐nearest neighbourhoods, Cubist) to account for the relationships between vegetation and these covariates. Different machine‐learning approaches vary in terms of computational requirements, predictive power and interpretability; the use of an ensemble of machine‐learning tools allows an assessment to be made of the robustness of the predictions (Hengl et al., 2018; Heung et al., 2016). The prediction accuracy of this dataset at 1 km is ca 70% globally. The PNV map has 13 biomes in the area covered by the SMPDS and EMBSeCBIO databases. We amalgamated biomes that covered a limited area or occurred as disjunct patches (e.g. erect dwarf shrub tundra, cool evergreen needleaf forest) because they do not sample within‐biome pollen variability adequately. As a result, we defined nine biomes (Table 1) that are structurally distinctive and represent distinct parts of the climate space of the region: tundra (TUND), desert (DESE), graminoids with forbs (GRAM), evergreen needleleaf woodland (ENWD), xeric shrubland (XSHB), cold evergreen needleleaf forest (CENF), temperate malacophyll broadleaf forest (TEDE), cool mixed evergreen needleleaf and deciduous broadleaf forest (CMIX), warm‐temperate evergreen needleleaf and sclerophyll broadleaf forest (WTFS). Thus, graminoid and forb tundra, erect dwarf shrub tundra, low and high shrub tundra, and prostrate dwarf shrub tundra were amalgamated into a single tundra biome (TUND in Table 1) and the cool evergreen needleleaf forest was amalgamated into the cool mixed evergreen needleleaf and deciduous broadleaf forest (CMIX in Table 1).

**TABLE 1:** Description of the nine biomes used in the analyses. The table gives the code, the full biome name and a brief definition of the vegetation

Code	Biome name	Definition
TUND	Tundra	Open vegetation characterized by cold‐adapted forbs, sedges, graminoids and shrubs found in arctic and high alpine regions
DESE	Desert	Open or non‐vegetated landscape characterized by sporadic occurrence of drought‐adapted vegetation including succulents, graminoids, forbs and shrubs
GRAM	Graminoids with forbs	Grasslands dominated by graminoids and forbs
ENWD	Evergreen needleleaf woodland	Semi‐open vegetation characterized by evergreen conifer trees, with an understorey of graminoids, forbs and sedges
XSHB	Xeric shrubland	Vegetation dominated by tall drought‐tolerant or sclerophyll shrubs.
CENF	Cold evergreen needleleaf forest	Forest vegetation dominated by cold‐tolerant conifers
TEDE	Temperate deciduous malacophyll broadleaf forest	Forest vegetation dominated by summer green trees
CMIX	Cool mixed evergreen needle leaf and deciduous broadleaf forest	Forest vegetation characterized by a mix of temperate summer green trees and conifers
WTFS	Warm‐temperate evergreen needleleaf and sclerophyll broadleaf forest	Forest vegetation dominated by broad‐leaved and needle‐leaved evergreen trees, characteristic of Mediterranean‐type climates with summer drought

### 2.2 Biome characterization

The training and testing datasets were created by sub‐sampling the modern pollen samples using the PNV map to assign samples to biomes. We used samples from this larger area to sample the whole of the realized range of each biome and to include biomes that are not currently represented in the EMBSeCBIO region but may have occurred there under different climate conditions in the past. This ‘space‐for‐time’ substitution is warranted because the climatic drivers of plant compositional turnover across space are similar to those that drive compositional turnover through time during the Late Quaternary (Blois et al., 2013).

There are some locations with multiple modern samples in a very small area, for example, multiple moss polsters within a catchment of a few square kilometres. This could lead to over‐representation of some localities in the training dataset and therefore an apparent reduction in the variability of assemblages from the biome(s) involved. Similarly, there are some regions where there are modern pollen samples from sites that are geographically close together and have similar vegetation, resulting in over‐sampling of that biome in the training dataset (Figure S1) and consequently overfitting to the better sampled biome. To limit redundancy in such cases, a single modern sample at each point (defined by latitude and longitude coordinates) was randomly selected for the analyses. We tested different ways of subsampling the data to reduce the biome oversampling bias. The best performance was obtained by down‐sampling biomes with a large number of samples to create a dataset with a similar number of samples from each biome. The down‐sampled data were then split into training and test datasets.

The observed PNV biome for each pollen sample in the modern training dataset was derived using different search windows (from 12 × 12 km up to 50 × 50 km) around the location of the sample point. We determined both the dominant and subdominant biome in each search window for subsequent evaluation, based on which biomes occupied the largest (dominant) and second largest (sub‐dominant) number of 1 km^2^ pixels within the search window. We also tested the impact of using different proportions of sites for the training set and the test set for each of these search windows, splitting using ratios of 50:50, 60:40, 70:30, 75:25 and 80:20. We tested how well each of these combinations predicted the modern vegetation of the EMBSeCBIO region using only modern samples from this region in the test set. The best performance, based on the balanced accuracy, was obtained using a search window of 20 × 20 km centred on the sample location, with a training/test data partitioning ratio of 70:30 (Tables S2 and S3).

The training samples were grouped according to the dominant biome observed in the PNV map. Each modern biome was then characterized by the relative abundance (expressed in terms of the mean, and standard deviation) of all taxa present to account for variability in pollen abundances within each biome.

### 2.3 Calculation of the dissimilarity and similarity scores

We calculated the following coefficient of dissimilarity between the pollen assemblage of a sample from the test dataset and each biome:

(1) $$D_{\mathrm{ik}}=\sqrt{\sum_{\mathrm{jk}}\frac{{\left(p_{\mathrm{jk}}-\mu_{\mathrm{ji}}\right)}^{2}}{{\left(s_{\mathrm{ji}}+\varepsilon \right)}^{2}},}$$

where *D*_*ik*_ is the dissimilarity of pollen sample *k* from biome *i*; *p*_*jk*_ is the pollen percentage of taxon *j* in sample *k*; *μ_j_*_*i*_ is the mean of taxon *j* in biome *i*, *s*_*ji*_ is the sample standard deviation of taxon *j* in biome *i* and *ε* is a parameter. Summation is over all taxa in sample *k*.

Equation (1) is based on the idea that each taxon has a certain distribution of values within a biome, and more weight is assigned to those taxa for which this distribution is narrow. For each taxon, *D*_*ik*_ is related inversely to the log‐likelihood that a pollen sample is drawn from the population represented by the abundances of that taxon among the modern pollen samples in each biome. The term *ε* has two functions. First, it decreases the weight of characteristic taxa with low values of *s* (e.g. due to limited sampling) in the calculation of the dissimilarity measure. Second, it allows the absence of taxa from a fossil sample (*s* = 0) to be informative, with *ε* = 0 they could not be considered in the calculation. The choice of the value of *ε* represents a balance between accuracy and sensitivity, since small values of *ε* could result in more accurate predictions of well‐sampled biomes but increase the sensitivity such that poorly sampled biomes are less well predicted. Values between 0% and 1% were tried, and good results obtained with a value of 0.5% (Table S4) which was therefore used for the subsequent calculations.

The dissimilarity values were converted to similarities by:

(2) $$S_{\mathrm{ik}}=e^{-D_{\mathrm{ik}}/100},$$

where *S*_*ik*_ is an approximation of the likelihood of biome *i* given pollen sample *k*. By the nature of the dissimilarity index, these similarity scores quantify how close a pollen sample is to the mean abundance values in the biome, accounting for within‐biome variability. Samples that represent vegetation closer to the ecotonal boundary of a biome will necessarily have a lower similarity score, although this score will still be higher than the score for other biomes.

### 2.4 Evaluation of the modern biome reconstructions

The biome reconstructions were evaluated quantitatively using a matrix of predicted versus observed vegetation at each site (‘confusion matrix’: e.g. Bigelow et al., 2003). We constructed confusion matrices for the test dataset and the EMBSeCBIO dataset, based on both the dominant and subdominant biome registered in the search window around the sample. The confusion matrix provides an assessment of the accuracy of the reconstructions, but is strongly influenced by imbalances in the sampling of different classes (Carrillo et al., 2014; Grandini et al., 2020). Some biomes are less well represented in the training dataset even after down‐sampling to reduce differences in sample numbers between biomes. Under these circumstances, the ‘balanced accuracy’ metric, defined as the average accuracy obtained on all classes (Carrillo et al., 2014), provides a better performance metric. The balanced accuracy metric is given by:

(3) $$\mathrm{BA}=\frac{1}{l}\sum_{i=1}^{l}\frac{k_{i}}{n_{i}},$$

where *k*_*i*_ is the number of sites correctly predicted for biome *i*, *l* is the number of biomes and *n*_*i*_ is the number of sites in biome *i*. Evaluation was performed both on the testing dataset and on samples only from the EMBSeCBIO region. The reconstructed modern biome distribution for the EMBSeCBIO region was mapped.

We compared the performance of the new method to a reconstruction made using the biomisation method (Marinova et al., 2018). We reclassified the 13 biomes used by Marinova et al. (2018) to match the set of nine biomes (Table 1) used here, by considering the three types of warm‐temperate vegetation they recognized as equivalent to our WTFS biome, combining cool and cold evergreen needleleaf forest, and combining deciduous broadleaf woodland with evergreen needleleaf woodland. However, the allocations of taxa to PFTs and PFTs to these biomes were entirely based on Marinova et al. (2018) (Tables S5 and S6). We then applied the biomisation technique to samples from the EMBSeCBIO region. The accuracy of the reconstructed biomes was evaluated against the PNV map based on the dominant and subdominant biome in the search window.

### 2.5 Impact on the flickering switch problem

We used fossil pollen data from the EMBSeCBIO database to assess the impact of the new method on the stability of biome reconstructions through time. The EMBSeCBIO database contains 187 records covering some part of the Holocene. New age‐depth models have been produced for 149 of these records using the IntCal20 calibration curve (Reimer et al., 2020) and the ‘rbacon’ R package (Blaauw et al., 2021) in the framework of the ‘AgeR’ R package (Villegas‐Diaz et al., 2021), which automates the supervised creation of multiple age models for multiple cores. Optimum age modelling scenarios were initially selected from multiple ‘rbacon’ age models run using different prior accumulation rate and thickness values, using the lowest quantified area between the prior and posterior accumulation rate distribution curves; the optimum model was verified by comparing the distance of the estimated ages and the dating control points to check the accuracy of the model interpolation and through visual inspection to ensure that changes in accumulation rate were independent of the control points. We extracted 30 fossil pollen records with a temporal resolution <100 years and covering an interval of at least 1000 years, on the assumption that it is unlikely that there will be multiple biome changes within a specific interval between samples that are <100 years apart and that large numbers of such changes would indicate instability due to the flickering switch problem. We applied the biomisation technique and our new method to these 30 records and counted the number of biome changes that occurred between adjacent samples. This number was standardized for differing lengths of record by dividing by the total number of samples in the record minus one.

### 2.6 Estimation and evaluation of biome analogue thresholds

We used the similarity scores obtained from the pollen samples in the modern testing dataset for each biome (Figure S3) and made pairwise comparisons between biomes. We obtained the optimal threshold value differentiating each pair of biomes by calculating specificity and sensitivity metrics (Figure S4) and plotting these on a ROC curve (Figure S5) using the ‘cutpoint’ R package (Thiele, 2021), where the point with the maximum balance (sensitivity + specificity) was selected as the optimal threshold between two biomes. The ROC curve has been used previously in MAT‐based vegetation reconstructions to assess the ability of different distance metrics to distinguish between groups and to avoid ad hoc interpretations of dissimilarity scores and thresholds (Gavin et al., 2003). We derived a single threshold value per biome, as the lowest value obtained in comparisons of that biome against all other biomes. If a pollen sample has a similarity score below the lowest threshold value for all biomes, it is assumed to represent a non‐analogue assemblage. We tested this approach using (1) the 3650 modern samples in the testing dataset, and (2) a synthetic dataset of 70 samples, using pollen abundances from actual samples and removing taxa or assigning abundances to different taxa randomly. We then applied this approach to fossil samples to determine if it identified non‐analogue samples in these records and summarized these analyses by quantifying the proportion of records with at least one non‐analogue sample in specific time periods, defined as 200‐year windows with 50% overlap.

## 3 RESULTS

### 3.1 Within‐biome variability in the training dataset

**FIGURE 3:**
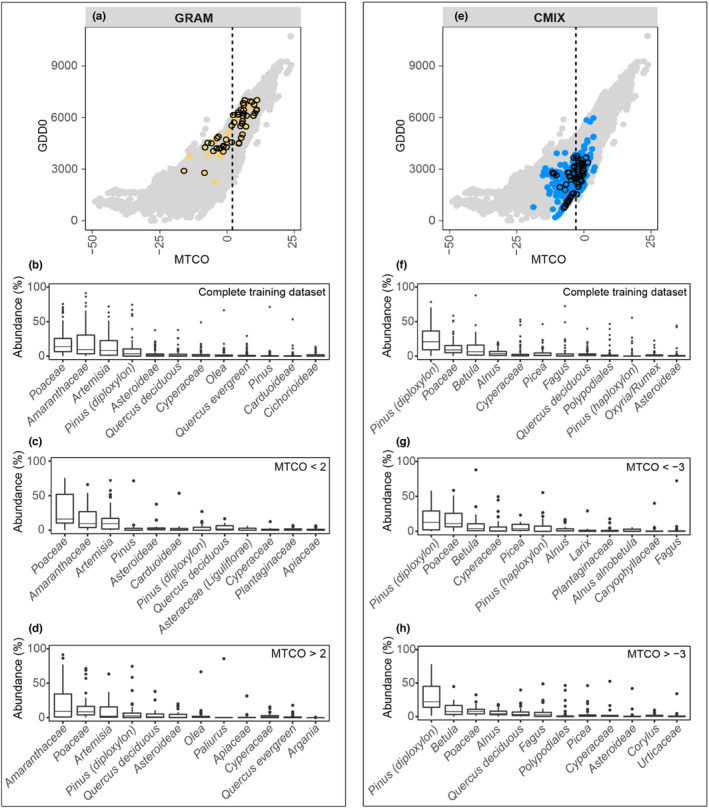
Examples of the characterization of individual biomes in terms of their pollen assemblages and the distribution of the pollen sites belonging to these biomes in bioclimatic space. The bioclimatic space is defined in terms of the mean temperature of the coldest month (MTCO) and growing degree days above a base level of 0°C (GDD0). The climate data at each pollen site were derived from Harrison (2020). Plot (a) shows the bioclimatic space occupied by graminoids with forbs (GRAM, yellow dots) and plot (e) the space occupied by cool mixed evergreen needleleaf and deciduous broadleaf forest (CMIX, blue dots) in the context of the biolcimatic space of the whole Europe and middle eastern region (grey dots). Dashed lines indicate MTCO mid‐point values for each biome. The box plots show the median and standard deviation of the abundance of the 12 most abundant individual taxa in GRAM and CMIX, where (b, f) show samples from the complete training dataset, (c, g) shows samples from the training set with MTCO values lower than the mid‐point values and (d, h) show samples from the training set with MTCO values higher than the mid‐point value. Box plots for the other biomes are given in supplementary.

Most biomes show considerable within‐biome variability in taxon abundances, reflecting changes in the importance of individual taxa along climate and environmental gradients (Figure 3; Figure S2). The most abundant taxa in every biome of the training dataset correspond to PFTs characteristic of that vegetation type (Figure 3). For example, open‐vegetation types such as GRAM (Figure 3b) are characterized by high abundances of Poaceae (*μ* ≈ 20%), Amaranthaceae (*μ* ≈ 20%), *Artemisia* (*μ* ≈ 15%), Asteroideae (*μ* ≈ 3%) and Cyperaceae (*μ* ≈ 3%). Similarly, CMIX (Figure 3f) is characterized by high abundances of *Pinus* (diploxylon) (*μ* ≈ 25%), *Betula* (*μ* ≈ 10%), *Alnus* (*μ* ≈ 5%), *Picea* (*μ* ≈ 4%), *Fagus* (*μ* ≈ 4%) and deciduous *Quercus* (*μ* ≈ 4%). Each biome has less characteristic taxa that represent understorey vegetation (e.g. Poaceae in CMIX, *μ* ≈ 11%), indicates azonal vegetation (e.g. Cyperaceae in CMIX, *μ* ≈ 5%) or (e.g. *Pinus* diploxylon in GRAM, *μ* ≈ 9%).

Differences in composition along the climate gradients covered by a specific biome are reflected in the abundance of the dominant taxa (Figures 3a,e). For example, Poaceae, *Artemisia* and Amaranthaceae are the three most important taxa in GRAM (Figure 3b) but their relative importance changes between cold and warm ends (here distinguished by the MTCO value that represents the mid‐point of the sample range, < or > 2°C for GRAM and < or > −3°C for CMIX) of the climate range occupied by the biome. Differences can be even more extreme for subordinate taxa. *Picea* and *Fagus* are present in GRAM in low abundances when MTCO <2°C (Figure 3c) but are absent when winter temperatures are higher, where the subordinate taxa include *Olea* and *Argania* in low abundance (Figure 3d). Similar shifts in composition can be seen in other biomes, for example CMIX (Figure 3f), where *Pinus* (Diploxylon) and Poaceae are the dominant taxa throughout the range, but *Betula* and *Picea* are important at the cold (Figure 3g) and *Quercus* and *Alnus* are important at the warm (Figure 3h) end.

### 3.2 Assessment of the modern reconstructions

The modern reconstruction using the training dataset reached a balanced accuracy of 66%, which improved to 76% when both dominant and subdominant biomes were considered (Table 2). The best predictions, considering both dominant and subdominant biomes (Table 2), were for CMIX (89%), TUND (83%), XSHB (82%), GRAM (76%), TEDE (74%), ENWD (72%), CENF (71%), DESE (70%). The least well‐predicted biome was WTFS (65%). Persistent mismatches can occur between closely related biomes: DESE samples were allocated to xerophytic shrublands in 13% of the cases, while samples from GRAM were allocated to DESE in 11% of the cases. Similarly, CENF samples were allocated to CMIX in 11% of cases.

**TABLE 2:** Quantitative comparison of predicted and observed dominant and sub‐dominant (in brackets) biomes across the whole testing dataset. The observed dominant and sub‐dominant biomes are taken from the Hengl et al. (2018) potential natural vegetation map. The biome codes are: CENF, cold evergreen needleleaf forest; CMIX, cool mixed evergreen needleleaf and deciduous broadleaf forest; DESE, desert; ENWD, evergreen needleleaf woodland; GRAM, graminoids with forbs; TEDE, temperate deciduous malacophyll broadleaf forest; TUND, tundra; WTFS, warm‐temperate evergreen needleleaf and sclerophyll broadleaf forest; XSHB, xeric shrubland. The total number of predicted and observed records for each biome are also shown (∑). The grey diagonal shows the number of correctly predicted samples, while off‐diagonal elements are those incorrectly predicted.

Biomes	Predicted									∑
	DESE	XSHB	WTFS	GRAM	ENWD	TEDE	CMIX	CENF	TUND	
Observed										
DESE	16[0]	3	1	2	1	0	0	0	0	23
XSHB	2	27[4]	1	4	0	0	0	0	0	38
WTFS	0	4	16[6]	4	0	3	1	0	0	34
GRAM	4	1	1	23[5]	1	1	1	0	0	37
ENWD	1	3	2	3	21[2]	0	0	0	0	32
TEDE	0	1	4	1	1	19[6]	2	0	0	34
CMIX	0	0	0	1	2	1	28[3]	0	0	35
CENF	0	0	0	1	1	2	4	23[2]	2	35
TUND	0	0	0	1	0	0	3	0	19[1]	24
∑	23	43	31	45	29	32	42	25	22	292

The modern reconstruction using the EMBSeCBIO dataset (Table 3) (excluding CENF and TUND which had too few samples for useful evaluation) reached a balanced accuracy of 64%, which improved to 77% when both the dominant and subdominant biomes were considered. The best predictions, considering both dominant and subdominant biomes (Table 3), were for DESE (89%), XSHB and ENWD (85%), GRAM (75%), TEDE (74%), and CMIX (67%). The least well‐predicted biome was WTFS (64%). GRAM samples were allocated to DESE in 12% of the cases, and CMIX samples were allocated to TEDE in 15% of the cases.

**TABLE 3:** Quantitative comparison of predicted and observed dominant and sub‐dominant (in brackets) biomes in the eastern Mediterranean‐Black Sea Caspian corridor region. The observed dominant and sub‐dominant biomes are taken from the Hengl et al. (2018) potential natural vegetation map. The biome codes are as follows: CENF, cold evergreen needleleaf forest; CMIX, cool mixed evergreen needleleaf and deciduous broadleaf forest; DESE, desert; ENWD, evergreen needleleaf woodland; GRAM, graminoids with forbs; TEDE, temperate deciduous malacophyll broadleaf forest; TUND, tundra; WTFS, warm‐temperate evergreen needleleaf and sclerophyll broadleaf forest; XSHB, xeric shrubland. The total number of predicted and observed records for each biome are also shown (∑). The grey diagonal shows the number of correctly predicted samples, while off‐diagonal elements are those incorrectly predicted.

Biomes	Predicted									∑
	DESE	XSHB	WTFS	GRAM	ENWD	TEDE	CMIX	CENF	TUND	
Observed										
DESE	44[7]	1	0	4	1	0	0	0	0	57
XSHB	1	7[10]	0	0	0	2	0	0	0	20
WTFS	0	22	137[14]	24	3	35	0	0	0	235
GRAM	12	3	0	61[12]	4	4	1	0	0	97
ENWD	1	3	5	8	87[28]	2	1	0	0	135
TEDE	12	10	15	11	24	199[29]	6	0	0	306
CMIX	0	0	1	1	9	10	36[9]	1	0	66
CENF	0	0	0	0	1	0	0	0	0	1
TUND	0	0	0	0	0	0	0	0	0	0
∑	77	56	172	121	157	281	52	1	0	917

**FIGURE 4:**
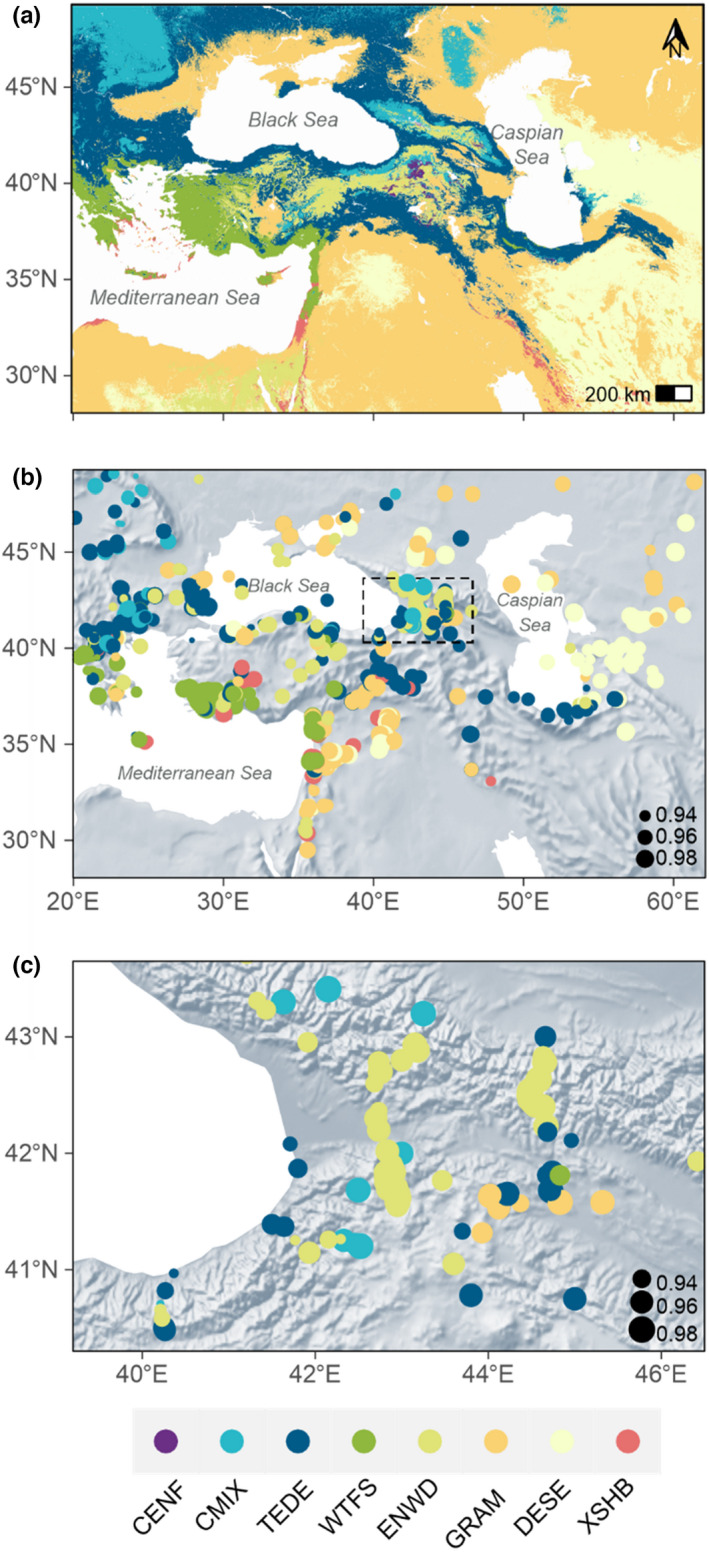
Maps showing (a) observed and (b) reconstructed biomes using the modern records across the eastern Mediterranean‐Black Sea Caspian corridor region. The colours show the observed or reconstructed biome. The biome codes are: CENF, cold evergreen needleleaf forest; CMIX, cool mixed evergreen needleleaf and deciduous broadleaf forest; DESE, desert; ENWD, evergreen needleleaf woodland; GRAM, graminoids with forbs; TEDE, temperate deciduous malacophyll broadleaf forest; TUND, tundra; WTFS, warm‐temperate evergreen needleleaf and sclerophyll broadleaf forest; XSHB, xeric shrubland. The size of the dots in (b) provides an indication of the similarity score obtained, where the larger dots mean higher similarities. Plot (c) shows an enlargement of the map of reconstructed biomes in the Caucasus region, where the distribution is strongly controlled by elevational gradients.

The method correctly predicted modern vegetation patterns across the EMBSeCBIO region (Figure 4), including the distribution of WTFS along the coastal plains around the Mediterranean and the transitions from forest through woodland to open vegetation in the Middle East and to the east of the Caspian Sea. The distribution of CMIX and TEDE in the Carpathian Mountains and the Balkans were also correctly predicted. In regions of complex topography, such as the Caucasus (Figure 4c), the method successfully captured the transition from forest to more open vegetation with elevation.

### 3.3 Impact of choice of training dataset

The construction of the training dataset had only a minor impact on the overall accuracy of the reconstructions: the average balanced accuracy was 76 ± 1.87% (Table S2). In evaluation of different biome search windows with different training/test splits, the average balanced accuracy was 76 ± 0.96% (Table S3). The prediction accuracy in the EMBSeCBIO region was similar (average balanced accuracy 76 ± 0.77%) to that for datasets covering all of the SMPDS region (Table S3). The removal of lakes larger than 50 km^2^, which have a larger pollen source area than the area used to allocate dominant and sub‐dominant biomes for each sample (Prentice, 1985; Sugita, 1993), improved the prediction, but only marginally from a balanced accuracy of 76% to 77%.

### 3.4 Comparison with reconstructions using the biomisation method

The balanced accuracy obtained for the EMBSeCBIO region using the new method was better than that obtained using the biomisation method (excluding CENF and TUND) (Table 4), with a value of 64% (compared to 49%) when considering only the dominant biome, and 76% (compared to 65%) when considering both dominant and sub‐dominant biomes. Both methods produced better predictions for open vegetation types than forests: DESE (89%), XSHB (85%) and ENWD (85%) are the best predicted biomes with the new method, and ENWD (89%), DESE (83%) and GRAM (73%) with biomisation. However, there is a considerably larger range of prediction accuracy with the biomisation approach. Thus, the worst predicted biome using the new method had an accuracy of 64% (WTFS), whereas the worst predicted biome using biomisation had an accuracy of only 37% (TEDE). Mismatches occur between similar biomes in both methods. Some GRAM samples, for example, were allocated to DESE in 12% of cases under the new method, and 15% of cases in biomisation. Similarly, CMIX samples were allocated to TEDE in 15% of cases using the new method and to CENF in 26% of cases using biomisation. Some TEDE samples were wrongly allocated either to ENWD (8% in the new method, 20% in biomisation) or WTFS (5% in the new method, 22% in biomisation).

**TABLE 4:** Quantitative comparison of predicted and observed dominant and sub‐dominant (in brackets) biomes in the eastern Mediterranean‐Black Sea Caspian corridor region. The predicted dominant and sub‐dominant biomes were obtained using the biomisation method as described by Marinova et al. (2018); the observed dominant and sub‐dominant biomes are taken from the Hengl et al. (2018) potential natural vegetation map. The biome codes are as follows: CENF, cold evergreen needleleaf forest; CMIX, cool mixed evergreen needleleaf and deciduous broadleaf forest; DESE, desert; ENWD, evergreen needleleaf woodland; GRAM, graminoids with forbs; TEDE, temperate deciduous malacophyll broadleaf forest; TUND, tundra; WTFS, warm‐temperate evergreen needleleaf and sclerophyll broadleaf forest; XSHB, xeric shrubland. The total number of predicted and observed records for each biome are also shown (∑). The grey diagonal shows the number of correctly predicted samples, while off‐diagonal elements are those incorrectly predicted.

Biomes	Predicted									∑
	DESE	XSHB	WTFS	GRAM	ENWD	TEDE	CMIX	CENF	TUND	
Observed										
DESE	43[15]	2	8	1	1	0	0	0	0	70
XSHB	1	4[3]	1	0	2	0	0	0	0	11
WTFS	1	0	127[29]	27	9	3	3	28	3	230
GRAM	14	1	5	47[19]	3	0	1	0	0	90
ENWD	1	0	3	10	90[80]	2	1	3	0	190
TEDE	9	0	55	31	50	81[12]	4	10	0	252
CMIX	2	0	3	10	6	0	21[10]	19	2	73
CENF	0	0	0	1	0	0	0	0	0	1
TUND	0	0	0	0	0	0	0	0	0	0
∑	86	10	231	146	241	98	40	60	5	917

### 3.5 Reconstruction stability

Down‐core reconstructions for high‐resolution records using the new method showed generally greater stability than was obtained using the biomisation approach. There was no difference in the range of the proportion of changes between contiguous samples per thousand years (0%–77%), but the new method reduced the proportion of changes in 70% of the records, and produced a similar number of changes in 7% of the records (Table S7). Thus, the new method provides more stable reconstructions and reduces the tendency for small variations in taxon abundance to cause unrealistic temporal shifts between biomes.

### 3.6 Assessment of the approach for non‐analogue detection

**FIGURE 5:**
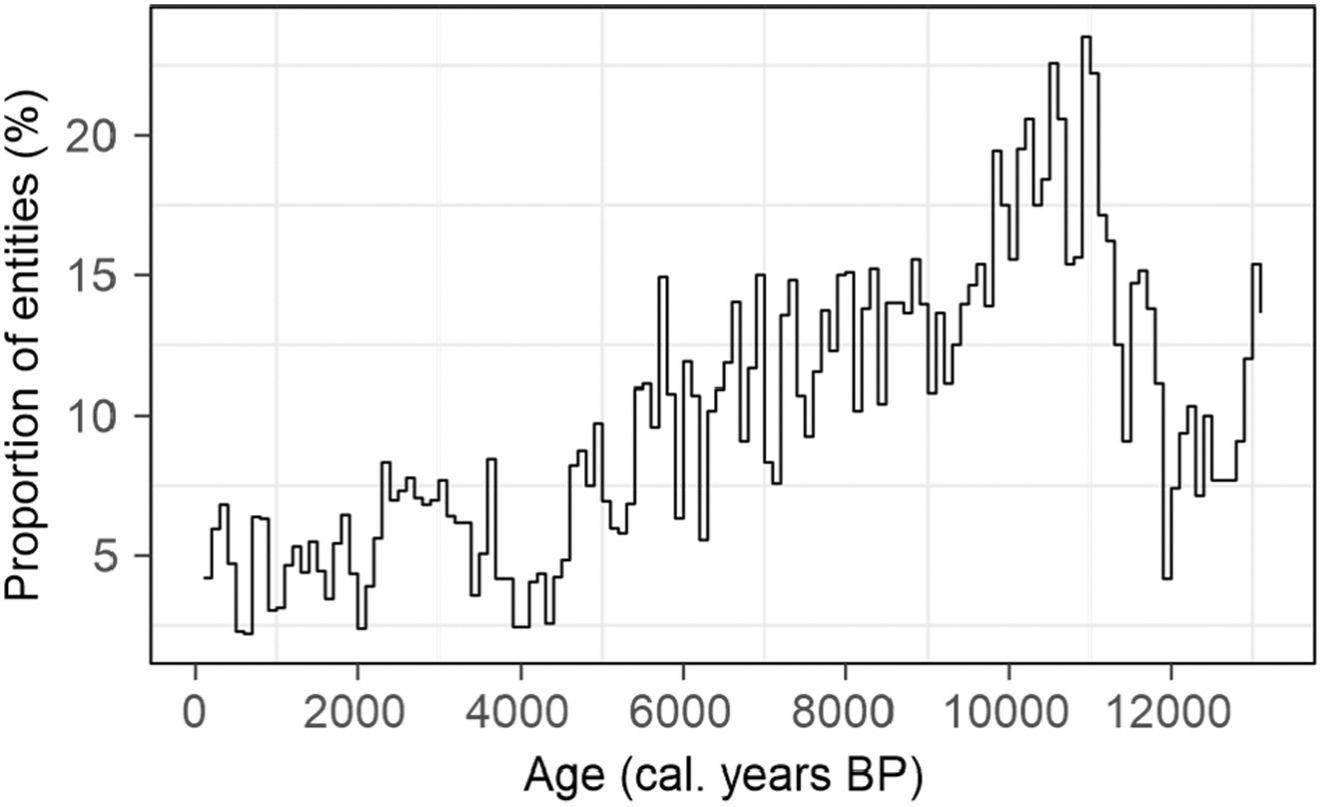
Proportion of high‐resolution pollen records (entities) having at least one sample identified as non‐analogue in a 200‐year time‐window over the past 12,000 years, where the time windows were constructed with 50% overlap. Values <5% are generally assumed to be false positives, but values above this are thought to indicate genuine non‐analogue vegetation assemblages.

A threshold value for identifying analogue and non‐analogue samples was estimated for each biome (Table S8). Only 4.5% of the modern samples were identified as non‐analogue, a value that is reasonable for false positives. However, 100% of the synthetic dataset were identified as non‐analogue. Application of this approach to the fossil dataset showed an abrupt increase in the proportion of non‐analogues at the transition between the late‐glacial and early Holocene (~11 ky), where the number of entities identified as having non‐analogue assemblages exceeds 20% (Figure 5). After ~4 ky, the proportion of entities identified as having non‐analogue samples is around 5% and taken to indicate the presence of false positives.

## 4 DISCUSSION

We have devised a method to reconstruct vegetation that is conceptually simple and uses the observed within‐biome variability in taxon abundances explicitly. Exploiting information about within‐biome variability avoids subjective choices about taxon allocations to PFTs and biomes inherent in biomisation. It also circumvents issues associated with the under‐ or over‐representation of some species in pollen assemblages. The new method produced a more accurate reconstruction of modern vegetation patterns in the EMBSeCBIO region than biomisation (77% vs. 65%) because it preserves the observed community properties acquired from all taxa across all samples. The substantial increase in balanced accuracy (64%–77%) when both dominant biome and subdominant biomes were considered shows, moreover, that many of the remaining misallocations occurred between climatically related biomes—a feature that is common to biomisation reconstructions (Prentice et al., 1996, 2000). The method identifies even poorly sampled biomes successfully: DESE is represented by far fewer samples than forest biomes in the training set, for example, but is predicted with 70% accuracy whereas scarcity of samples led to a very low probability of reconstructing DESE using biomisation, for example in Africa and the Arabian Peninsula (Jolly et al., 1998).

Our approach exploits a large modern pollen dataset to characterize biomes. This is important because previous research (Turner et al., 2021) has shown that the accuracy of pollen‐based climate reconstructions is contingent on maximizing the sampled environmental space and is unaffected by the inclusion of climatically insensitive or systematically overrepresented taxa. Turner et al. (2021) also showed that the range and continuity of sampling along an environmental gradient is more important than the sampling density, consistent with our decision to reduce over‐sampling of some biomes and eliminate redundant samples from the modern samples prior to model development. However, subsequent methodological decisions, such as the size of the search window or the partitioning of the data for testing and training, had very little impact and changed the overall accuracy of the reconstructions by less than ±2%.

The new method provides more stable reconstructions of past vegetation than the biomisation approach. Given that multiple switches between biomes within a limited period of time in high‐resolution pollen records are more likely a reflection of the over‐sensitivity of the reconstruction technique to minor fluctuations in taxon abundances rather than multiple changes in vegetation on decadal to sub‐centennial timescales, this suggests that the new method largely overcomes the so‐called flickering switch problem. Previous attempts to reconstruct vegetation patterns through time (e.g. Williams et al., 2000, 2011; Zanon et al., 2018) have minimized the flickering switch problem by focusing on reconstructions for widely spaced time periods. Our new approach opens up the possibility of making robust reconstructions at higher temporal resolution.

We have demonstrated how the similarity scores for individual biomes can be used to determine whether a given vegetation assemblage is present in the modern vegetation. We show that this approach identifies synthetic samples composed of extant taxa in combinations not seen in the modern dataset as non‐analogue. If we assume a 5% cut‐off for false positives based on the assignments of the modern testing dataset, there are analogues for late Holocene vegetation records from the EMBSeCBIO region but the number of non‐analogues samples during the late glacial and early Holocene exceeds 20%. This is consistent with other studies that have identified late glacial and early Holocene non‐analogue pollen assemblages in many regions (Caballero‐Rodríguez et al., 2017; Correa‐Metrio et al., 2012; Jones et al., 2017; Williams & Jackson, 2007), including the EMBSeCBIO region (Connor & Kvavadze, 2009).

Pseudobiomisation has been used to reconstruct LCCs for the Anatolian and Balkan parts of the EMBSeCBIO region (Fyfe et al., 2015; Woodbridge et al., 2014). The main focus of this work was to identify different degrees of anthropogenic modification of the vegetation (Fyfe et al., 2010), and most of the LCCs specifically include agricultural cover (e.g. mixed open vegetation, LCC8 which is defined as a combination of heath/scrubland, pastures/natural grassland and arable land). Although three LCCs are primarily natural vegetation, and are broadly equivalent to the biomes used in this analysis, they represent only ⪅25 sites and this precludes a direct comparison between the new method and the pseudobiomisation approach. Nevertheless, the overall accuracy of the pseudobiomisation for the whole of Europe was only 50%, and predictions for the Mediterranean region were recognized as being poor (Fyfe et al., 2015; Woodbridge et al., 2014), suggesting that the new method is likely to be more accurate.

We have evaluated our reconstructions using a PNV map created using a machine‐learning approach with pollen‐based biome reconstructions to infer the vegetation that should be present as a function of climate and other environmental factors from the evidence available about the (relict) natural vegetation. Although this map is reasonably accurate globally, it is nevertheless most reliable where there are abundant data for training and less reliable where data are scarce (Hengl et al., 2018). However, the stability of our reconstructions using different modern sub‐samples drawn from different locations suggests that uncertainties in the PNV mapping have had little influence on our reconstructions.

Although we have focused on the reconstruction of natural vegetation, the new method could be applied to reconstruct actual vegetation cover including land use. This would be useful to quantify the magnitude of human impact on vegetation through time and the effect of such changes on biodiversity, landscape degradation, carbon storage and climate. However, while there are many satellite‐based sources of high‐resolution land‐cover data (Buchhorn et al., 2020; CCI Land Cover, 2017; Gong et al., 2013; Sulla‐Menashe et al., 2019), they focus on vegetation properties that impact land‐surface feedbacks rather than biomes. Thus, for example, they make no distinction between the broadleaved evergreen tree cover characteristic of tropical environments and the sclerophyll broadleaved evergreen trees of the Mediterranean. Furthermore, since the amount of bare ground is important for water‐exchange with the atmosphere, they distinguish low cover areas regardless of whether this cover is herbs, shrubs or trees. Finally, field validation of these maps has been limited, where it has been done the accuracy is only about 80% (Tsendbazar et al., 2021). Nevertheless, although there would be a considerable amount of work involved in creating more accurate biome descriptions from existing land cover datasets, it could be a useful to explore this in future.

A more important limitation of our method may be the use of a universal search window (20 × 20 km) to determine the biome for each modern site, selected by comparison of performance scores for different windows. The increase in balanced accuracy from 66% to 76% when both the dominant and subdominant biomes are considered suggests a greater degree of precision to take account of the pollen source area of each site could be beneficial. A fifth of the modern samples are from lakes or bogs (19%), and 68% of these records are from basins <1 km^2^ in area. The selected search window is close to the theoretical pollen source area for small (<1 km^2^) basins (Prentice, 1988; Sugita, 1993). Of the remaining modern samples, 33% are from moss polsters and 16% are from soil or other surface materials, both categories that record an even more local signal than small lakes or bogs, although the area sampled depends on vegetation openness and may be comparable with small lakes in some settings (Beer et al., 2007; Bunting et al., 2004; Jackson & Wong, 1994; Prentice, 1988). This again suggests that the choice of a 20 × 20 km search window is reasonable. Improvements to prediction accuracy could be achieved using a search window appropriate to the pollen source of each site. However, the EMBSeCBIO database lacks information about site type for 27% and about basin size for 14% of the sample records. Furthermore, basin size is only recorded categorically, so the quantitative information required to allow us to test this idea still needs to be collected.

Our new method combines the relative simplicity of the biomisation approach with the more rigorous statistical basis of dissimilarity‐based reconstruction techniques. One major advantage of the current approach compared to existing dissimilarity‐based reconstruction techniques is that it explicitly takes account of within‐biome variability. Samples that represent ecotonal transitions between biomes can therefore be assigned to a biome and the nature of the ecotone represented can be determined by comparing the scores for other biomes, providing a more nuanced way of examining past vegetation changes and transitions.

## 5 CONCLUSIONS

Our new approach provides a promising way of reconstructing past vegetation taking account of the non‐proportionality in the relationship between vegetation cover and pollen abundances. There are alternative approaches to doing this, but their accuracy is tempered by very high data demands. Our new method requires an extensive modern pollen dataset but is less data‐demanding than other approaches and yields robust results. Biomisation depends on expert judgement for the assignment of taxa to PFTs and PFTs to biomes; our method avoids this limitation, while producing more accurate and temporally stable reconstructions.

## CONFLICT OF INTEREST

The authors declare no conflict of interest.

## BIOSKETCH

**Esmeralda Cruz‐Silva** is a PhD candidate at the University of Reading. Her thesis is on the interactions of climate, vegetation and human activities in the circum‐Mediterranean region. She is currently a member of the SPECIAL group focused on the reconstruction and analysis of past climate and terrestrial environmental changes (https://research.reading.ac.uk/palaeoclimate/). All the authors of this study share an interest in vegetation responses to climate change and the consequences of those responses.

**Author Contributions**: SPH, ICP, EM and ECS designed this study. ICP, SPH and ECS developed the reconstruction technique. EM, SPH and ECS expanded and revised the EMBSeCBIO database, including the construction of new age models. ECS and SPH were responsible for the analysis. ECS and SPH wrote the first draft of the paper, and all authors contributed to the final version.

## Supporting information


Appendix S1.

## Data Availability

The SMPDS data are available through the University of Reading Data archive at: https://researchdata.reading.ac.uk/194/. The EMBSeCBIO pollen data base is available through the University of Reading Data archive at: https://researchdata.reading.ac.uk/309/. The code for the modern and fossil reconstructions in this paper is available at: https://github.com/esmeraldacs/Intra_biome_variation_method.
