# Supplementary material for: A new method based on surface‐sample pollen data for reconstructing palaeovegetation patterns
Source: J Biogeogr. 2022 Jun 15;49(7):1381–96. doi: 10.1111/jbi.14448 (PMC9328394; doi:10.1111/jbi.14448)
Supplement: Supplementary file 1 — Appendix S1. [file JBI-49-1381-s001.docx]

Journal of Biogeography

**SUPPORTING INFORMATION**

A new method based on surface-sample pollen data for reconstructing palaeovegetation patterns.

Esmeralda Cruz-Silva^1^, Sandy P. Harrison^1^, Elena Marinova^2^, I. Colin Prentice^3^

1: School of Archaeology, Geography & Environmental Science, Reading University, Whiteknights, Reading, RG6 6AH, UK

2: Laboratory for Archaeobotany, Baden-Württemberg Cultural Heritage State Office, Fischersteig 9, 78343 Geienhofen-Hemmenhofen, Germany

3: Georgina Mace Centre for the Living Planet, Department of Life Sciences, Imperial College London, Silwood Park Campus, Buckhurst Road, Ascot SL5 7PY, UK

This supplementary contains the following figures and tables:

Supplementary Figure 1: Number of modern pollen samples allocated to each biome. The biomes were derived from the Potential Natural Vegetation (PNV) Map of Hengl et al. (2018) using a search window of 20 km x 20 km. This figure illustrates the extremely uneven nature of the sampling of biomes in the combined SPECIAL Modern Pollen Data Set (SMPDS) and the Eastern Mediterranean-Black Sea Caspian Corridor (EMBSeCBIO) database used for the training and testing datasets, and hence the necessity for down-weighting the representation of some biomes. The biome codes are: CMIX: cool mixed evergreen needleleaf and deciduous broadleaf forest, WTFS: warm-temperate evergreen needleleaf and sclerophyll broadleaf forest, TEDE: temperate deciduous malacophyll broadleaf forest, CENF: cold evergreen needleleaf forest, XSHB: xeric shrubland, GRAM: graminoids with forbs, TUND: tundra, ENWD: evergreen needleleaf woodland, and DESE: desert.

Supplementary Figure 2: Box and whisker plots showing the 25 most abundant taxa in each biome. The boxes show the median and standard deviation of the abundance of individual taxa, the whiskers show the 85% confidence interval, and the open circles show outliers. The biomes are: tundra (TUND), desert (DESE), graminoids with forbs (GRAM), evergreen needleleaf woodland (ENWD), xeric shrubland (XSHB), cold evergreen needleleaf forest (CENF), temperate malacophyll broadleaf forest (TEDE), cool mixed evergreen needleleaf and deciduous broadleaf forest (CMIX), warm-temperate evergreen needleleaf and sclerophyll broadleaf forest (WTFS).

Supplementary Figure 3. Density distribution of similarity scores to every biome for samples assigned graminoids with forbs (GRAM) and cool mixed evergreen needleleaf and deciduous broadleaf forest (CMIX) according to the Potential Natural Vegetation (PNV) Map of Hengl et al. (2018). The biomes are: tundra (TUND), desert (DESE), graminoids with forbs (GRAM), evergreen needleleaf woodland (ENWD), xeric shrubland (XSHB), cold evergreen needleleaf forest (CENF), temperate malacophyll broadleaf forest (TEDE), cool mixed evergreen needleleaf and deciduous broadleaf forest (CMIX), warm-temperate evergreen needleleaf and sclerophyll broadleaf forest (WTFS). These plots are used to derive similarity score thresholds to determine samples with potential non-analogue vegetation types.

Supplementary Figure 4. Measurement of the balance between sensitivity and specificity in the evaluation of possible cut-off points between two biomes for samples assigned to graminoids with forbs (GRAM) and cool mixed evergreen needleleaf and deciduous broadleaf forest (CMIX) according to the Potential Natural Vegetation (PNV) Map of Hengl et al. (2018). The similarity score comparisons are made between GRAM and tundra (TUND) and between CMIX and xeric shrubland (XSHB). The dotted lines show the cut-off point above which the sample would be allocated to GRAM and CMIX respectively.

Supplementary Figure 5: Optimal threshold detection. Comparison of the distribution of similarity scores for samples allocated to (a) graminoids with forbs (GRAM) and (c) cool mixed evergreen needleleaf and deciduous broadleaf forest (CMIX) according to the Potential Natural Vegetation (PNV) Map of Hengl et al. (2018). The scores for GRAM are compared to scores obtained on these samples for tundra (TUND) and the scores for CMIX are compared to scores obtained on these samples for xeric shrubland (XSHB). The Receiver Operating Characteristic (ROC) curve for (b) the GRAM/TUND and (d) the CMIX/XSHB paired comparisons show the optimal threshold (black dot) in the ROC curve and as a dashed line in the density curves. The area under the ROC curve (AUC) is a measure of the overall ability of the optimal threshold to differentiate between the two biomes being compared. The AUC ranges between 0.5 (ROC curve is a diagonal line – no discrimination between categories as both density distributions are identical) and 1 (ROC curve follow left and upper borders of the ROC graph – perfect discrimination as the density distributions are completely separated).

Supplementary Table 1: Allocation of pollen taxa found in samples of the Eastern Mediterranean-Black Sea Caspian Corridor (EMBSeCBIO) pollen database into the 247 taxa represented in the SPECIAL Modern Pollen Data Set (SMPDS) (Harrison, 2019; Wei et al., 2020). The table indicates the taxonomic level of aggregation, the name used for the amalgamated taxon, and the component species or genera included in this taxon.

Supplementary Table 2: Comparison of the quality of reconstructions based on training datasets constructed in different ways for the modern data set for the whole of the SPECIAL Modern Pollen Data Set (SMPDS) and for the data set encompassing only the Eastern Mediterranean-Black Sea Caspian Corridor (EMBSeCBIO) region. Assessments are made on the accuracy with respect to only the dominant biome and to the dominant and sub-dominant biomes identified in a 20 x 20 km^2^ search window around each sampling point according to the Potential Natural Vegetation (PNV) Map of Hengl et al. (2018). We give both the accuracy and the balanced accuracy metrics for each split of the training and testing datasets.

Supplementary Table 3: Comparison of the quality of reconstructions based on using different areas around each sample to determine the observed vegetation type and on using different training and testing data partitioning ratios. The size of the search window is given in km. For the ratio of the size of the training and testing data sets, a value of 70:30 means that 70% of the data are used as the training set and 30% of the data are used as the test set. Assessments are made on the accuracy with respect to only the dominant biome and to the dominant and sub-dominant biomes identified in each search window around each sampling point according to the Potential Natural Vegetation (PNV) Map of Hengl et al. (2018) for the modern data set for the whole of the SPECIAL Modern Pollen Data Set (SMPDS) and for the data set encompassing only the Eastern Mediterranean-Black Sea Caspian Corridor (EMBSeCBIO) region. We give both the accuracy and the balanced accuracy metrics for each assessment.

Supplementary Table 4. Comparison of the quality of reconstructions for the modern data set for the whole of the SPECIAL Modern Pollen Data Set (SMPDS) and for the data set encompassing only the Eastern Mediterranean-Black Sea Caspian Corridor (EMBSeCBIO) region based on using different values for ε in equation 1 (range from 0.01 to 1). Assessments are made on the accuracy with respect to only the dominant biome and to the dominant and sub-dominant biomes identified in a 20 x 20 km^2^ search window around each sampling point according to the Potential Natural Vegetation (PNV) Map of Hengl et al. (2018). We give both the accuracy and the balanced accuracy metrics for assessment.

Supplementary Table 5: Plant functional types (PFTs) included in each biome in the biomisation procedure. The allocations of PFTs to biomes follows Marinova et al. (2018) but has been modified to take account of the amalgamation of some biomes in our analyses.

Supplementary Table 6: Taxon allocation to plant functional types (PFTs) used in the biomisation reconstructions. The allocations of taxa to PFTs es follows Marinova et al. (2018) but has been modified to take account of the amalgamation of some biomes in our analyses.

Supplementary Table 7: Comparison of percent changes in biomes between adjacent samples for selected high-resolution Holocene pollen records using the new method and the standard biomisation approach. The biome with the lowest percentage of changes is highlighted in bold.

Supplementary Table 8: Optimal threshold for each biome used for detection of potential non-analogue assemblages.

Supplementary Figure 1: Number of modern pollen samples allocated to each biome. The biomes were derived from the Potential Natural Vegetation (PNV) Map of Hengl et al. (2018) using a search window of 20 km x 20 km. This figure illustrates the extremely uneven nature of the sampling of biomes in the combined SPECIAL Modern Pollen Data Set (SMPDS) and the Eastern Mediterranean-Black Sea Caspian Corridor (EMBSeCBIO) database used for the training and testing datasets, and hence the necessity for down-weighting the representation of some biomes. The biome codes are: WTFS: warm-temperate evergreen needleleaf and sclerophyll broadleaf forest, CMIX: cool mixed evergreen needleleaf and deciduous broadleaf forest, TEDE: temperate deciduous malacophyll broadleaf forest, CENF: cold evergreen needleleaf forest, XSHB: xeric shrubland, ENWD: evergreen needleleaf woodland, GRAM: graminoids with forbs, DESE: desert, TUND: tundra.


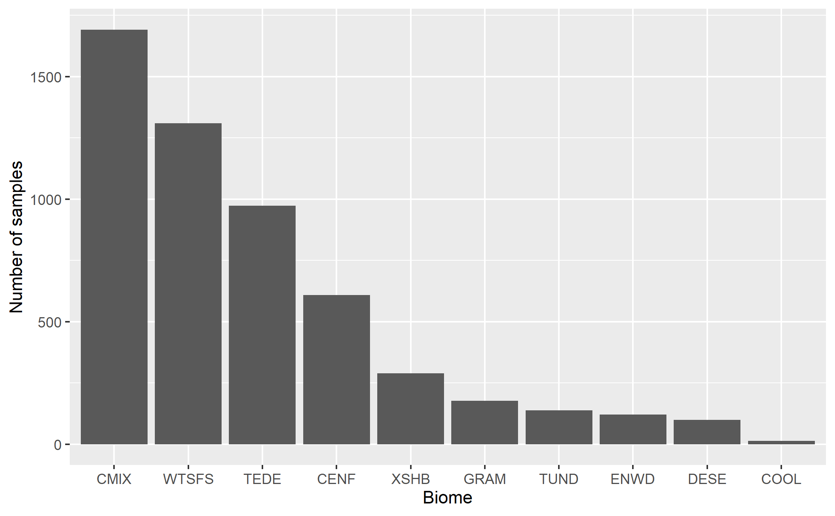


Supplementary Figure 2: Box and whisker plots showing the 25 most abundant taxa in each biome. The boxes show the median and standard deviation of the abundance of individual taxa, the whiskers show the 85% confidence interval, and the open circles show outliers. The biomes are: tundra (TUND), desert (DESE), graminoids with forbs (GRAM), evergreen needleleaf woodland (ENWD), xeric shrubland (XSHB), cold evergreen needleleaf forest (CENF), temperate malacophyll broadleaf forest (TEDE), cool mixed evergreen needleleaf and deciduous broadleaf forest (CMIX), warm-temperate evergreen needleleaf and sclerophyll broadleaf forest (WTFS).


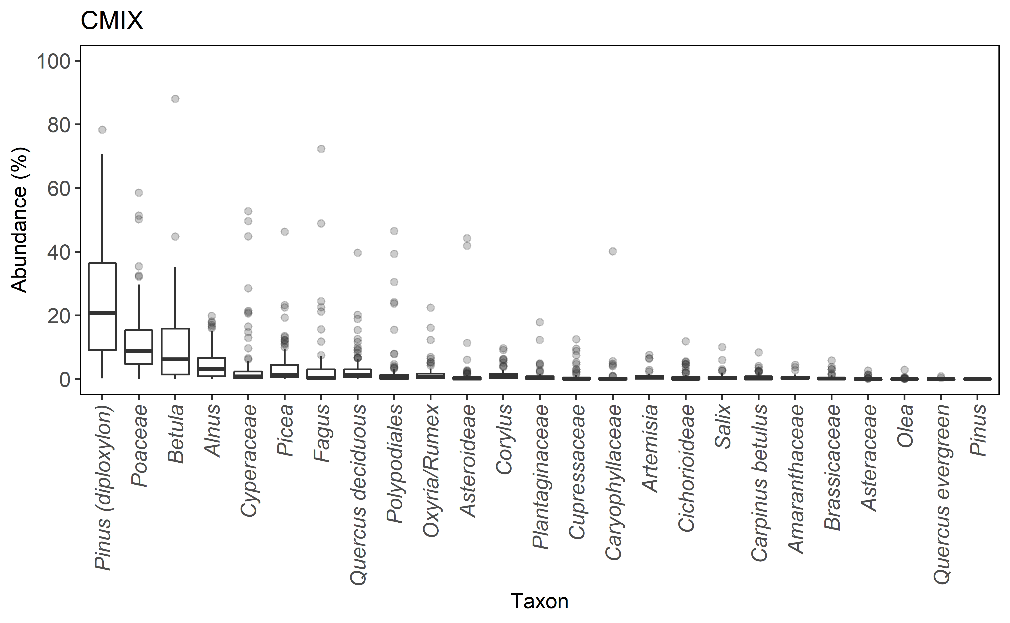


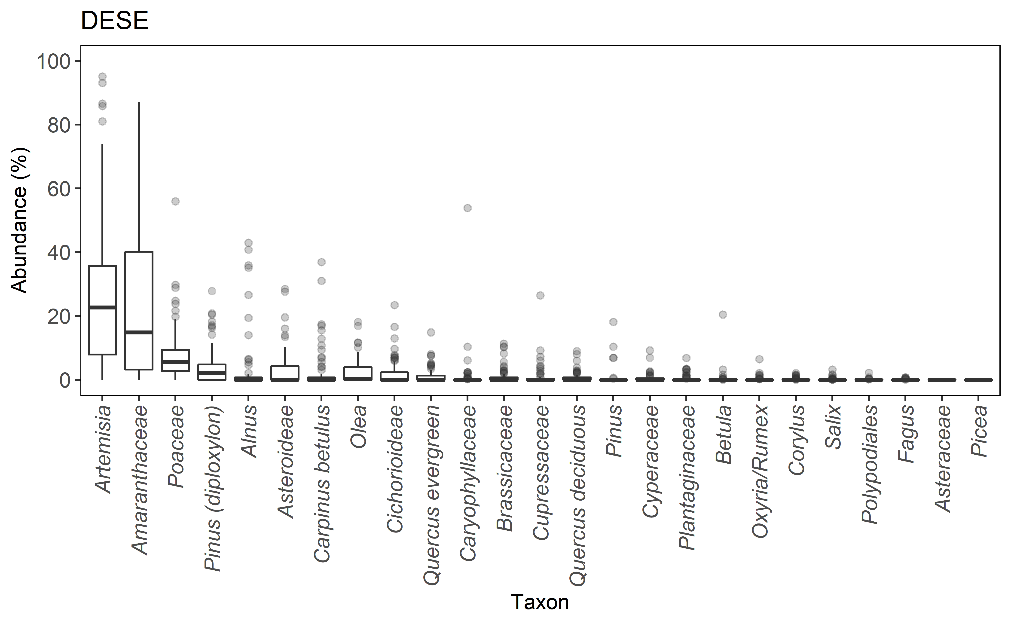


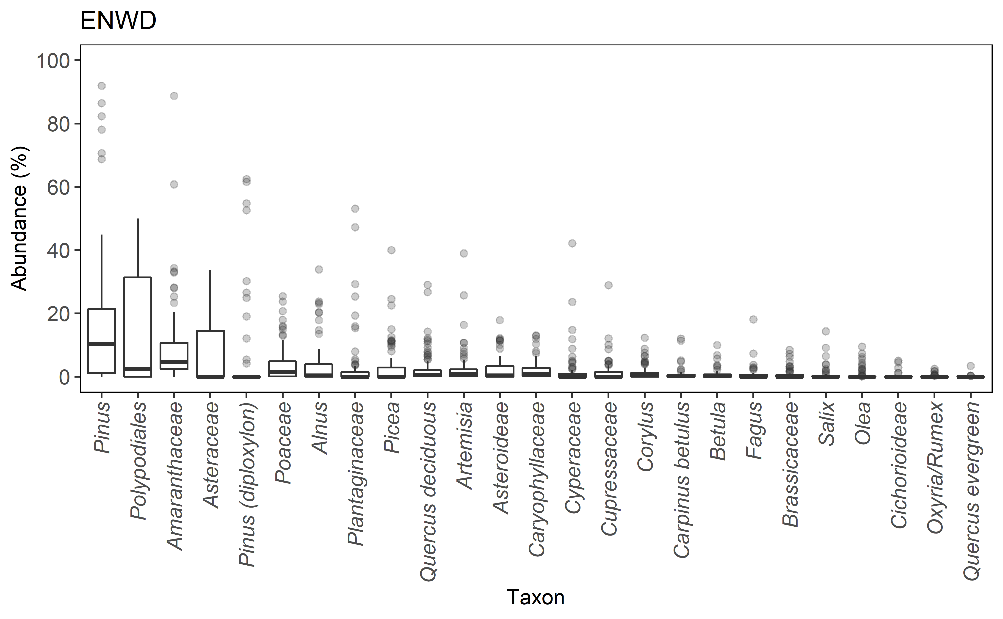


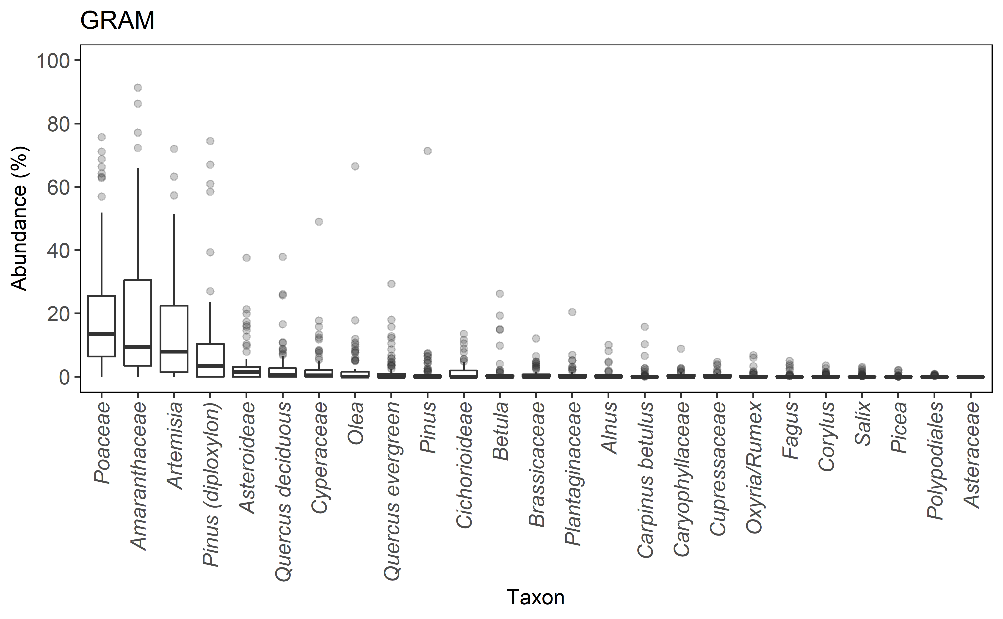


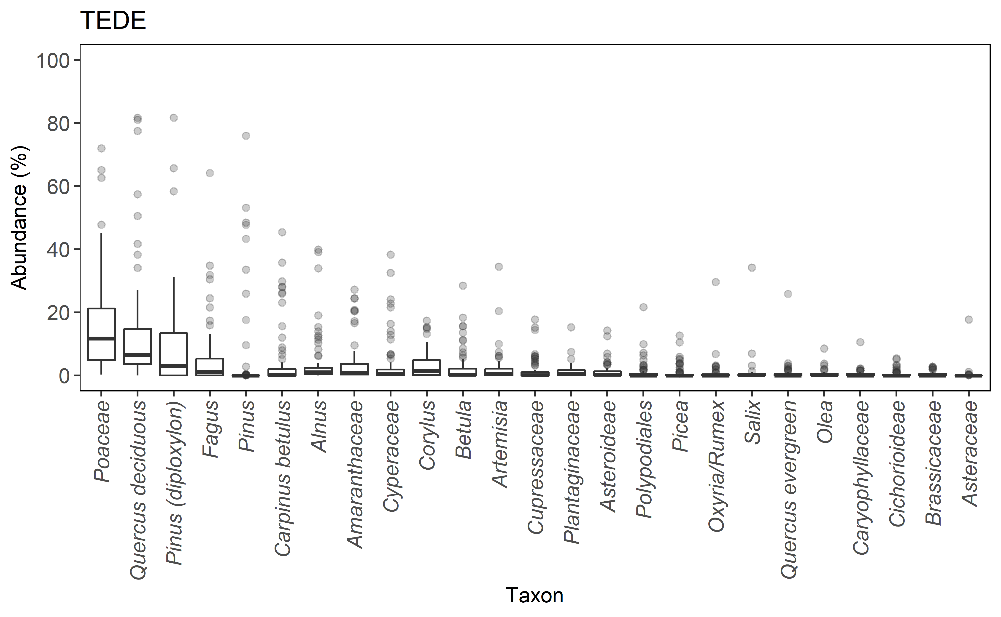


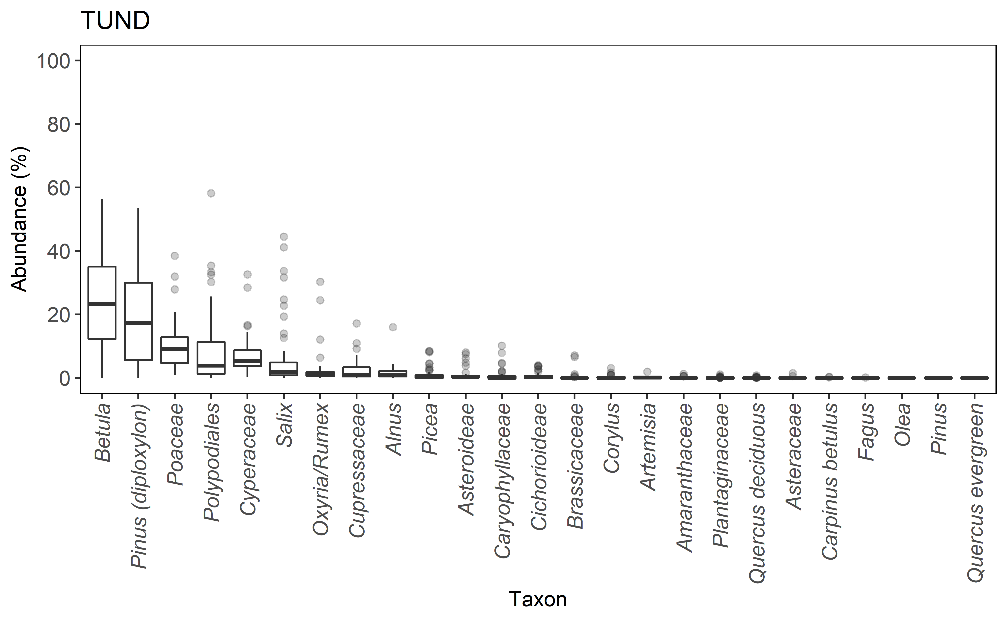


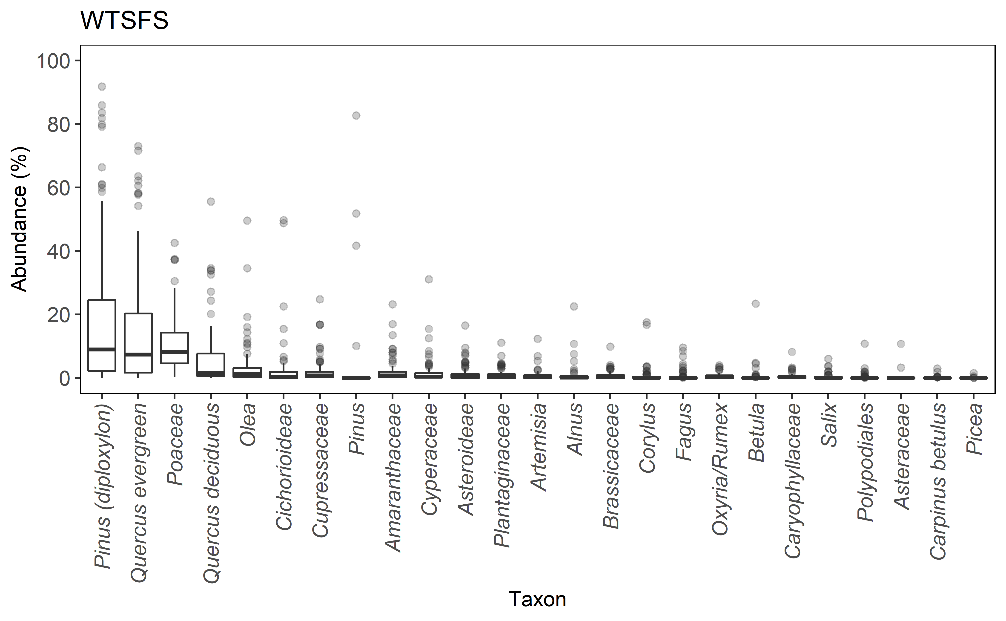


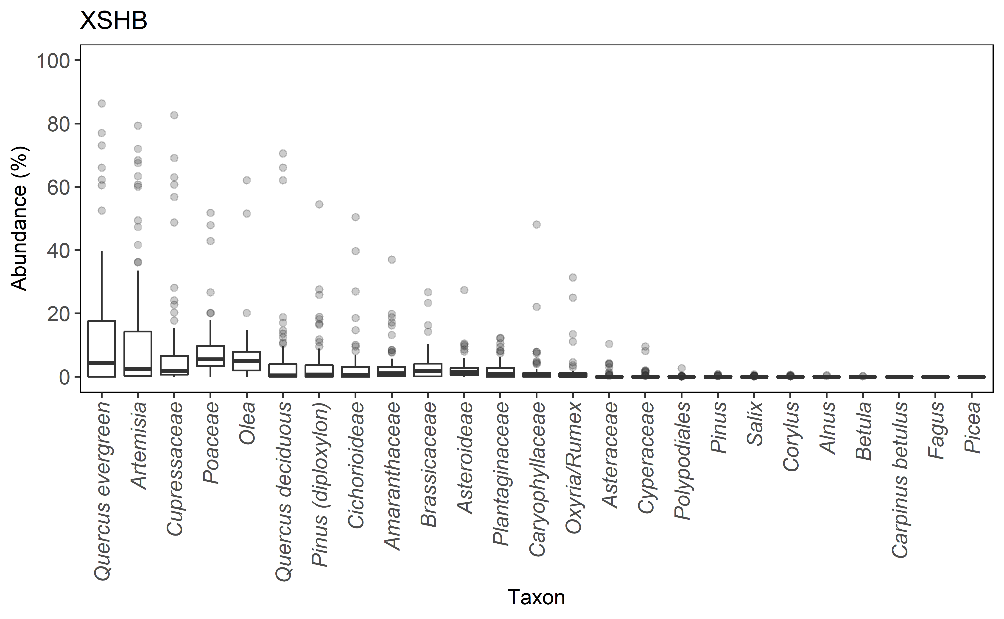


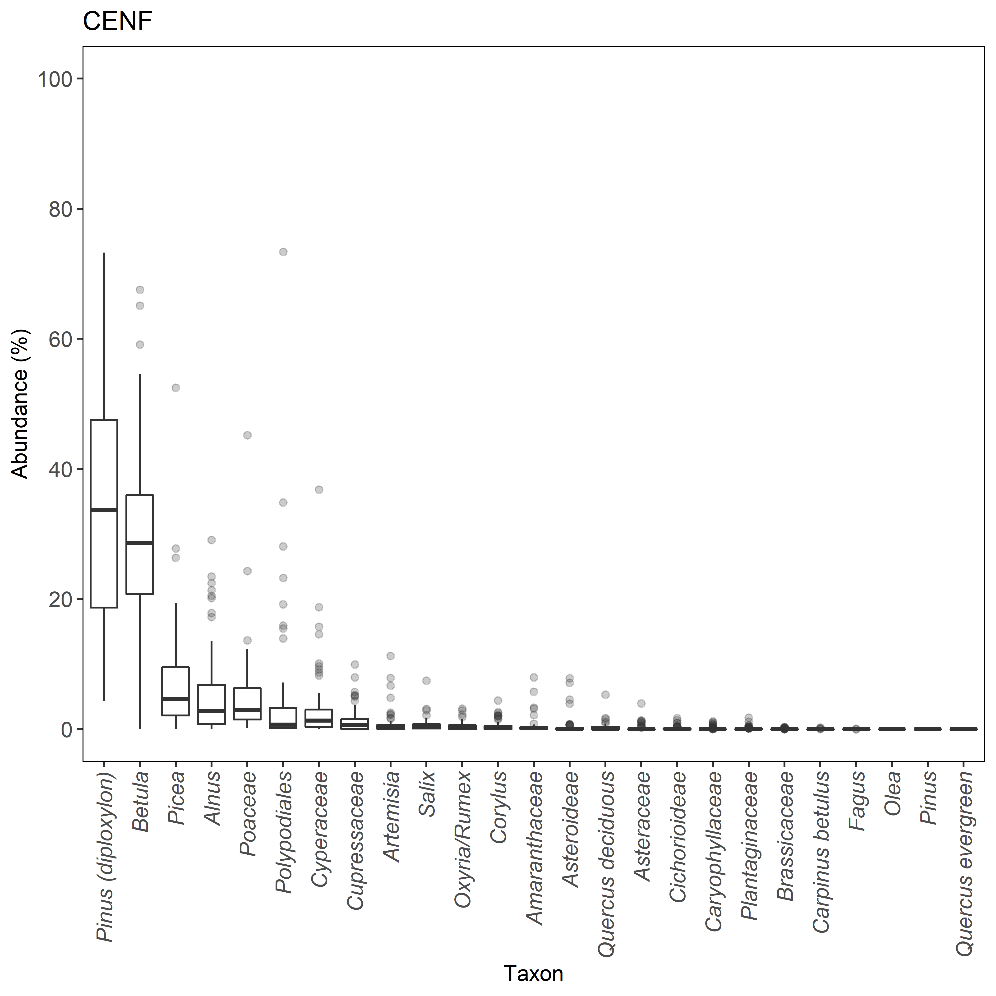


Supplementary Figure 3. Density distribution of similarity scores to every biome for samples assigned graminoids with forbs (GRAM) and cool mixed evergreen needleleaf and deciduous broadleaf forest (CMIX) according to the Potential Natural Vegetation (PNV) Map of Hengl et al. (2018). The biomes are: tundra (TUND), desert (DESE), graminoids with forbs (GRAM), evergreen needleleaf woodland (ENWD), xeric shrubland (XSHB), cold evergreen needleleaf forest (CENF), temperate malacophyll broadleaf forest (TEDE), cool mixed evergreen needleleaf and deciduous broadleaf forest (CMIX), warm-temperate evergreen needleleaf and sclerophyll broadleaf forest (WTFS). These plots are used to derive similarity score thresholds to determine samples with potential non-analogue vegetation types.


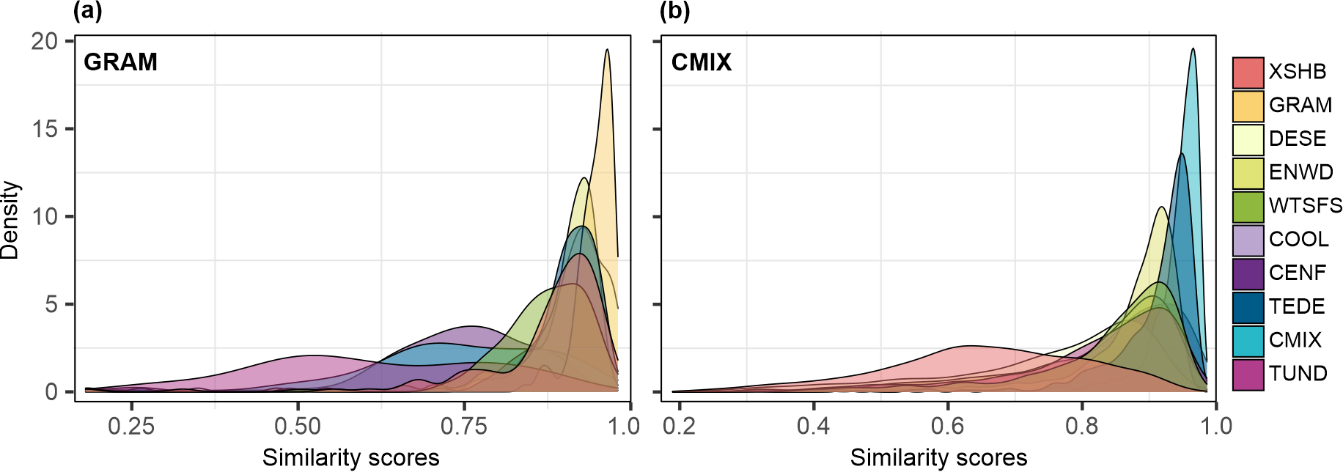


Supplementary Figure 4. Measurement of the balance between sensitivity and specificity in the evaluation of possible cut-off points between two biomes for samples assigned to graminoids with forbs (GRAM) and cool mixed evergreen needleleaf and deciduous broadleaf forest (CMIX) according to the Potential Natural Vegetation (PNV) Map of Hengl et al. (2018). The similarity score comparisons are made between GRAM and tundra (TUND) and between CMIX and xeric shrubland (XSHB). The dotted lines show the cut-off point above which the sample would be allocated to GRAM and CMIX respectively.


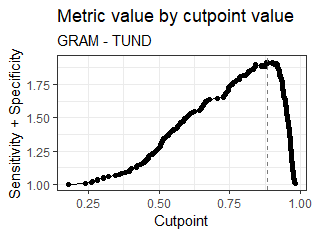

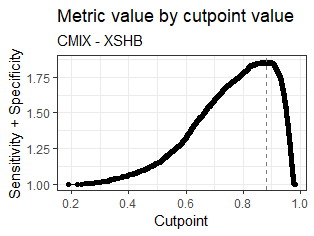


Supplementary Figure 5: Optimal threshold detection. Comparison of the distribution of similarity scores for samples allocated to (a) graminoids with forbs (GRAM) and (c) cool mixed evergreen needleleaf and deciduous broadleaf forest (CMIX) according to the Potential Natural Vegetation (PNV) Map of Hengl et al. (2018). The scores for GRAM are compared to scores obtained on these samples for tundra (TUND) and the scores for CMIX are compared to scores obtained on these samples for xeric shrubland (XSHB). The Receiver Operating Characteristic (ROC) curve for (b) the GRAM/TUND and (d) the CMIX/XSHB paired comparisons show the optimal threshold (black dot) in the ROC curve and as a dashed line in the density curves. The area under the ROC curve (AUC) is a measure of the overall ability of the optimal threshold to differentiate between the two biomes being compared. The AUC ranges between 0.5 (ROC curve is a diagonal line – no discrimination between categories as both density distributions are identical) and 1 (ROC curve follow left and upper borders of the ROC graph – perfect discrimination as the density distributions are completely separated).


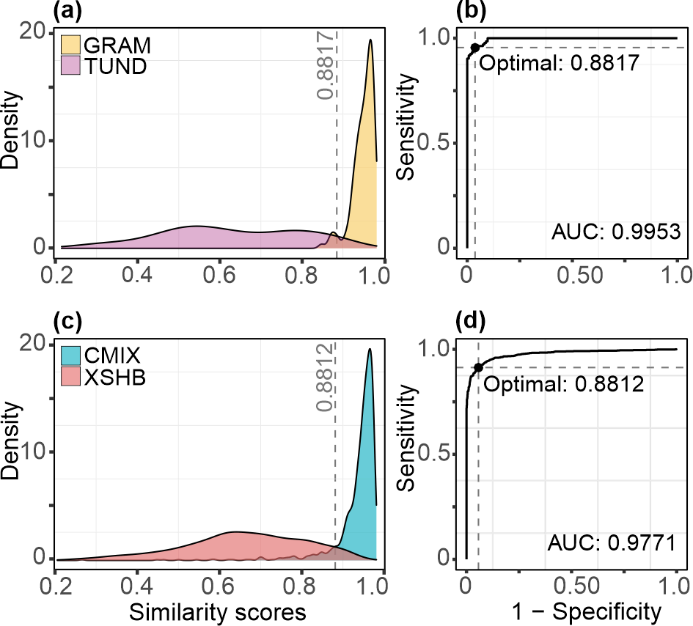


Supplementary Table 1: Allocation of pollen taxa found in samples of the Eastern Mediterranean-Black Sea Caspian Corridor (EMBSeCBIO) pollen database into the 247 types represented in the SPECIAL Modern Pollen Data Set (SMPDS) (Harrison, 2019; Wei et al., 2020). The table indicates the taxonomic level of aggregation, the name used for the amalgamated taxon, and the component species or genera included in this taxon.

| **Taxonomic level** | **Amalgamated taxon name** | **Individual taxa included** |
| --- | --- | --- |
| Genus | *Abies* | *Abies, Abies alba, Abies cilicica, Abies nordmanniana, Abies pinsapo subsp marocana* |
| Family | Acanthaceae | *Acanthus mollis* |
| Genus | *Acer* | *Acer, Acer campestre, Acer campestre* type*, Acer pseudoplatanus, Acer* type |
| Genus | *Aconitum* | *Aconitum, Aconitum group, Aconitum napellus* type*, Aconitum septentrionale, Aconitum* type |
| Family | Actinidiaceae | *Actinidia* |
| Genus | *Adonis* | *Adonis, Adonis aestivalis* type*, Adonis* type |
| Family | Adoxaceae | *Adoxa, Adoxa moschatellina, Adoxa* type |
| Genus | *Aesculus* | *Aesculus, Aesculus hippocastanum* |
| Genus | *Ailanthus* | *Ailanthus* |
| Family | Aizoaceae | *Carpobrotus* |
| Genus  sub-group | *Alnus* | *Alnus, Alnus glutinosa, Alnus glutinosa* type*, Alnus glutinosa/Alnus incana, Alnus incana, Alnus incana* type*, Alnus incana/Alnus cordata* type*, Alnus non-viridis, Alnus* type |
| Genus  sub-group | *Alnus alnobetula* | *Alnus viridis* |
| Family | Amaranthaceae | *Aellenia* type*,* Amaranthaceae*, Amaranthus, Atriplex nudicaulis, Chenopodium, Chenopodium album, Halothamnus t*ype*, Noaea* type |
| Family | Amaryllidaceae | Amaryllidaceae*, Narcissus* |
| Family | Anacardiaceae | Anacardiaceae |
| Genus | *Andromeda* | *Andromeda* |
| Family | Apiaceae | *Aegopodium, Anthriscus sylvestris* type*, Anthriscus* type*,* Apiaceae*, Apium, Apium* type*, Astrantia, Astrantia* type*, Athamanta cretensis, Berula erecta* type*, Bunium* type*, Aegopodium podagraria, Bupleurum, Bupleurum* type*, Carum carvi, Chaerophyllum, Chaerophyllum hirsutum* type*, Chaerophyllum* type*, Conopodium, Conopodium majus, Daucaceae, Daucus carota, Ammi* type*, Daucus carota* type*, Daucus* type*, Echinophora, Eryngium, Eryngium ilicifolium, Eryngium* type*, Falcaria* type*, Ferula, Ferula* type*, Heracleum, Angelica, Heracleum laciniatum* type*, Heracleum sphondylium, Heracleum* type*, Laserpitium latifolium* type*, Laserpitium prutenicum, Ligusticum mutellina, Malabaila, Meum, Meum athamanticum, Neogaya simplex* type*, Angelica archangelica, Oenanthe, Oenanthe* type*, Orlaya, Orlaya grandiflora, Pastinaca* type*, Peucedanum, Peucedanum ostruthium, Peucedanum* type*, Pimpinella, Pimpinella major* type*, Angelica* type*, Pimpinella* type*, Pleurospermum austriacum, Sanicula, Sanicula europaea, Sanicula type, Scandix, Seseli* type*, Torilis, Torilis arvensis, Turgenia* type*, Anisosciadium* type*, Anthriscus, Anthriscus sylvestris* |
| Genus | *Aquilegia* | *Aquilegia* type |
| Family | Araceae | Araceae |
| Genus | *Arbutus* | *Arbutus, Arbutus* type*, Arbutus unedo* |
| Genus | *Arctostaphylos* | *Arctostaphylos, Arctostaphylos uva-ursi* |
| Genus | *Argania* | *Argania spinosa* |
| Family | Aristolochiaceae | *Aristolochia* |
| Genus | *Artemisia* | *Artemisia, Artemisia genipii/Artemisia mutellina, Artemisia herba-alba* type*, Artemisia* type*, Artemisia vulgaris* type |
| Family | Asclepiadaceae | Asclepiadaceae |
| Family | Asparagaceae | Asparagaceae*, Asparagus, Asparagus* type*, Convallaria, Convallaria* type*, Maianthemum bifolium, Maianthemum* type*, Ornithogalum type, Scilla* type |
| Family | Asphodelaceae | *Eremurus* |
| Family | Asphodeliaceae | *Asphodeline, Asphodelus, Asphodelus albus, Asphodelus albus* type*, Asphodelus fistulosus* type*, Asphodelus* type |
| Family | Asteraceae | Asteraceae |
| Family  sub-group | Asteraceae (Liguliflorae) | Asteraceae (Liguliflorae) |
| Family  sub-group | Asteroideae | *Achillea, Antennaria, Antennaria* type*, Anthemis, Anthemis* type*, Arnica montana, Aster, Aster bellidiastrum, Aster* type*, Aster/Achillea, Aster/Achillea* type*, Achillea* type*,* Asteraceae (Tubuliflorae)*,* Asteroideae*, Bellis, Bellis* type*, Bidens, Bidens* type*, Calendula, Calendula* type*, Chrysanthemum alpinum, Doronicum, Achillea/Anthemis* type*, Erigeron, Eupatorium, Eupatorium* type*, Filago* type*, Filifolium sibiricum, Gnaphalium, Gnaphalium* type*, Helianthus, Helianthus* type*, Homogyne, Achillea/Aster, Homogyne alpina, Inula, Inula* type*, Logfia* type*, Matricaria* type*, Petasites, Petasites* type*, Senecio, Senecio* type*, Solidago, Adenostyles* type*, Solidago* type*, Solidago virgaurea* type*, Tussilago farfara, Tussilago* type*, Xanthium, Xanthium spinosum, Xanthium strumarium, Xanthium* type*, Ambrosia, Ambrosia artemisiifolia* type*, Ambrosia* type*, Ambrosia/Xanthium* |
| Genus | *Astragalus* | *Astragalus, Astragalus alpinus* type*, Astragalus* type |
| Family | Berberidaceae | Berberidaceae |
| Genus | *Berberis* | *Berberis, Berberis vulgaris, Mahonia* |
| Genus | *Betula* | *Betula, Betula* type*, Betula alba, Betula alba* type*, Betula pendula, Betula pendula/Betula pubescens, Betula pubescens, Betula pubescens* type*, Betula sect. Albae, Betula tortuosa* |
| Genus | *Betula (Chamaebetula)* | *Betula fruticosa, Betula humilis/Betula nana, Betula nana, Betula nana* type |
| Family | Boraginaceae | *Alkanna, Cynoglossum creticum, Echium, Echium* type*, Echium vulgare, Heliotropium* type*, Lithospermum, Myosotis, Myosotis arvensis* type*, Myosotis* type*, Onosma, Anchusa, Pulmonaria, Pulmonaria* type*, Symphytum, Symphytum* type*, Anchusa arvensis, Anchusa type,* Boraginaceae*, Borago officinalis, Cerinthe, Cerinthe minor, Cerinthe type* |
| Family | Brassicaceae | *Arabidopsis, Cardamine pratensis* type*, Cardamine* type*, Crambe, Descurainia, Draba, Draba type, Hornungia* type*, Lepidium* type*, Matthiola, Sinapis, Barbarea* type*, Sinapis* type*, Brassica, Brassica* type*,* Brassicaceae*,* Brassicaceae type*, Capsella bursa-pastoris* type*, Capsella* type*, Cardamine* |
| Genus | *Bruckenthalia* | *Bruckenthalia* |
| Genus | *Buxus* | *Buxus, Buxus sempervirens* |
| Genus | *Calluna* | *Calluna, Calluna* type*, Calluna vulgaris, Calluna vulgaris* type |
| Family | Campanulaceae | *Campanula, Phyteuma, Phyteuma* type*, Campanula* type*,* Campanulaceae*, Jasione, Jasione montana, Jasione* type*, Legousia, Lobelia dortmanna, Lobelia* type |
| Family | Capparaceae | Capparidaceae*, Capparis, Capparis spinosa, Capparis* type |
| Family | Caprifoliaceae | Caprifoliaceae*, Scabiosa columbaria* type*, Scabiosa rotata* type*, Scabiosa* type*, Scabiosa/Succisa* type*, Succisa, Succisa pratensis, Succisa pratensis* type*, Succisa* type*, Dipsacaceae, Dipsacus, Dipsacus fullonum* type*, Dipsacus* type*, Knautia, Knautia arvensis, Knautia* type*, Scabiosa* |
| Genus | *Carduoideae* | *Arctium, Centaurea, Centaurea collina, Centaurea collina* type*, Centaurea cyanus, Centaurea cyanus* type*, Centaurea depressa* type*, Centaurea jacea, Centaurea jacea* type*, Centaurea montana, Centaurea montana* type*, Arctium* type*, Centaurea nigra, Centaurea nigra* type*, Centaurea rhenana* type*, Centaurea scabiosa, Centaurea scabiosa* type*, Centaurea solstitialis, Centaurea solstitialis* type*, Centaurea* type*, Cirsium, Cirsium* type*, Arctium/Jurinea, Cirsium/Carduus, Cirsium/Gundelia, Cousinia, Echinops, Gundelia* type*, Jurinea* type*, Onopordum, Onopordum* type*, Saussurea, Saussurea alpina, Carduoideae, Saussurea* type*, Serratula, Serratula* type*, Carduus, Carduus* type*, Carlina, Carlina* type*, Carthamus* |
| Genus | *Carpinus betulus* | *Carpinus, Carpinus betulus, Carpinus betulus* type*, Carpinus* type |
| Genus | *Carpinus orientalis/Ostrya* | *Carpinus orientalis, Carpinus orientalis* type*, Carpinus orientalis/Ostrya, Carpinus orientalis/Ostrya carpinifolia, Carpinus orientalis/Ostrya carpinifolia* type*, Carpinus/Ostrya, Carpinus/Ostrya* type*, Ostrya, Ostrya* type |
| Family | Caryophyllaceae | *Agrostemma githago, Cerastium cerastioides* type*, Cerastium fontanum* type*, Cerastium* type*, Cerastium/Stellaria* type*, Corrigiola, Dianthus, Dianthus superbus* type*, Dianthus* type*, Frankenia, Frankenia* type*, Agrostemma* type*, Gypsophila, Gypsophila fastigiata, Gypsophila repens* type*, Gypsophila* type*, Herniaria, Herniaria glabra, Herniaria* type*, Herniaria/Paronychia, Illecebrum, Loeflingia, Arenaria, Lychnis, Lychnis flos-cuculi, Lychnis flos-cuculi* type*, Lychnis* type*, Lychnis viscaria* type*, Minuartia, Minuartia rubra* type*, Minuartia* type*, Minuartia verna* type*, Moehringia* type*, Arenaria* type*, Paronychia, Paronychia type, Polycarpon, Sagina, Sagina procumbens, Sagina* type*, Saponaria, Scleranthus, Scleranthus annuus, Scleranthus perennis, Caryophyllaceae, Scleranthus* type*,* Silenaceae*, Silene, Silene acaulis, Silene acaulis* type*, Silene alba* type*, Silene dioica* type*, Silene latifolia, Silene rupestris, Silene* type*,* Caryophyllaceae subfam*.* Silenoideae*, Silene vulgaris type, Spergula, Spergula arvensis, Spergula* type*, Spergularia, Spergularia* type*, Stellaria, Stellaria holostea, Stellaria holostea* type*, Stellaria nemorum, Cerastium, Stellaria nemorum* type*, Stellaria palustris, Stellaria* type*, Vaccaria* type*, Cerastium alpinum* type*, Cerastium arvense* type |
| Genus | *Cassiope* | *Cassiope, Cassiope* type |
| Genus | *Castanea* | *Castanea, Castanea sativa* |
| Genus | *Cedrus* | *Cedrus, Cedrus atlantica, Cedrus atlantica* type*, Cedrus libani* |
| Family | Celastraceae | Celastraceae*, Parnassia, Parnassia palustris* |
| Genus | *Celtis* | *Celtis, Celtis australis* |
| Genus | *Ceratonia* | *Ceratonia, Ceratonia siliqua, Ceratonia* type |
| Genus | *Cercis* | *Cercis siliquastrum* |
| Genus | *Chamaedaphne* | *Chamaedaphne, Chamaedaphne calyculata* |
| Genus | *Chamaerops* | *Chamaerops* |
| Genus | *Chimaphila* | *Chimaphila umbellata* |
| Genus | *Cichorioideae* | *Cichorioideae, Lactuca sativa* type*, Lactuca* type*, Leontodon helveticus, Leontodon* type*, Scorzonera humilis* type*, Scorzonera* type*, Sonchus* type*, Taraxacum, Taraxacum* type*, Cichorium, Cichorium intybus* type*, Cichorium* type*, Crepis, Crepis aurea, Crepis* typ*e, Hieracium* type*, Lactuca* |
| Family | Cistaceae | Cistaceae*, Halimium/Tuberaria, Tuberaria* |
| Genus | *Cistus* | *Cistus, Cistus salviifolius* type*, Cistus* type*, Cistus villosus* type*, Cistus albidus* type, *Cistus incanus, Cistus ladanifer, Cistus ladanifer* type*, Cistus monspeliensis* type*, Cistus populifolius* type*, Cistus salvifolius, Cistus salviifolius* |
| Genus | *Clematis* | *Clematis, Clematis* type*, Clematis vitalba* type |
| Genus | *Clethra* | *Clethra* |
| Family | Clusiaceae | *Guttiferae* |
| Family | Colchicaceae | *Colchicum, Colchicum autumnale* type*, Colchicum* type*, Colchicum/Merendera, Merendera* |
| Genus | *Colutea* | *Colutea* |
| Family | Convolvulaceae | *Calystegia, Calystegia sepium,* Convolvulaceae*, Convolvulus, Convolvulus arvensis, Convolvulus arvensis* type*, Corema album* type*, Cuscuta, Cuscuta europaea* type |
| Genus | *Coriaria* | *Coriaria, Coriaria myrtifolia* |
| Genus | *Cornus* | Cornaceae*, Cornus, Cornus mas, Cornus mas/Cornus suecica, Cornus sanguinea, Cornus suecica* |
| Genus | *Corylus* | *Corylus, Corylus avellana, Corylus avellana* type*, Corylus maxima, Corylus* type |
| Genus | *Cotinus* | *Cotinus, Cotinus coggygria* |
| Genus | *Cotoneaster* | *Cotoneaster* |
| Family | Crassulaceae | *Crassula,* Crassulaceae*, Sedum, Sedum rosea, Sedum* type*, Sempervivum, Umbilicus, Umbilicus rupestris* type |
| Genus | *Crataegus* | *Crataegus, Crataegus* type |
| Family | Cucurbitaceae | Cucurbitaceae |
| Family | Cupressaceae | Cupressaceae*, Taxodiaceae, Taxodium, Cupressus, Cupressus type, Juniperus, Juniperus communis, Juniperus communis* type*, Juniperus excelsa/Juniperus oxycedrus, Juniperus sabina, Juniperus* type |
| Genus | *Cynomorium* | *Cynomorium* |
| Family | Cyperaceae | *Carex, Fimbristylis, Rhynchospora, Rhynchospora alba, Rhynchospora* type*, Schoenoplectus, Schoenoplectus lacustris ssp lacustris, Schoenoplectus* type*, Schoenus* type*, Scirpus, Scirpus lacustris* type*, Carex hirta type, Scirpus* type*, Trichophorum caespitosum, Carex* type*,* Cyperaceae*, Cyperus, Cyperus* type*, Eleocharis palustris, Eriophorum angustifolium/Eriophorum vaginatum, Eriophorum* type |
| Family | Cytinaceae | *Cytinus hypocistis* type |
| Genus | *Daphne* | *Daphne, Daphne gnidium, Daphne gnidium* type*, Daphne* type |
| Genus | *Datisca* | *Datisca* |
| Genus | *Delphinium* | *Delphinium* type |
| Family | Dennstaedtiaceae | *Pteridium, Pteridium aquilinum, Pteridium aquilinum type, Pteridium* type |
| Genus | *Diapensia* | *Diapensia, Diapensia lapponica* |
| Genus | *Dryas* | *Dryas, Dryas octopetala* |
| Genus | *Elaeagnus* | *Elaeagnus* |
| Genus | *Empetrum* | *Empetrum, Empetrum nigrum, Empetrum* type |
| Genus | *Ephedra* | *Ephedra, Ephedra major* type*, Ephedra* type*, Ephedra alata* type*, Ephedra distachya, Ephedra distachya* type*, Ephedra fragilis, Ephedra fragilis ssp campylopoda, Ephedra fragilis* type*, Ephedra fragilis var campylopoda, Ephedra major* |
| Genus | *Equisetum* | *Equisetum* |
| Genus | *Erica* | *Erica, Erica lusitanica* type*, Erica scoparia* type*, Erica tetralix, Erica tetralix* type*, Erica* type*, Erica umbellata* type, *Erica arborea, Erica arborea* type*, Erica australis, Erica australis* type*, Erica cinerea, Erica cinerea* type*, Erica erigena* type*, Erica lusitanica* |
| Family | Ericaceae | Ericaceae*, Ericales, Lecythis, Moneses, Orthilia, Phyllodoce, Pyrola* |
| Genus | *Euonymus* | *Euonymus, Euonymus europaeus* |
| Family | Euphorbiaceae | *Acalypha, Euphorbia, Euphorbia* type*,* Euphorbiaceae |
| Family | Fabaceae | Fabaceae*, Glycine* |
| Family | Fabaceae (herbs) | *Anthyllis, Lathyrus/Vicia, Lotus, Lotus corniculatus, Lotus corniculatus type, Lotus type, Lotus uliginosus, Medicago, Medicago littoralis* type*, Medicago sativa, Medicago* type*, Coronilla, Melilotus, Melilotus* type*, Onobrychis, Onobrychis* type*, Oxytropis, Phaseolus type, Trifolium, Trifolium alpinum* type*, Trifolium badium* type*, Trifolium montanum, Ebenus/Hedysarum, Trifolium pratense, Trifolium pratense* type*, Trifolium repens, Trifolium repens* type*, Trifolium spadiceum* type*, Trifolium* type*, Vicia, Vicia cracca, Vicia cracca* type*, Vicia faba* type*, Hedysarum hedysaroides, Vicia sylvatica* type*, Vicia* type*,* Viciaceae*, Hippocrepis comosa, Hippocrepis* type*, Lathyrus, Lathyrus* type*, Lathyrus/Vicia* |
| Genus | *Fagus* | *Fagus, Fagus orientalis, Fagus sylvatica* |
| Genus | *Ficus* | *Ficus, Ficus carica* |
| Genus | *Flueggea* | *Securinega tinctoria* type |
| Genus | *Frangula* | *Frangula, Frangula alnus* |
| Genus | *Fraxinus* | *Fraxinus, Fraxinus angustifolia, Fraxinus angustifolia subsp. oxycarpa, Fraxinus angustifolia* type*, Fraxinus excelsior, Fraxinus excelsior* type*, Fraxinus ornus, Fraxinus ornus* type*, Fraxinus oxycarpa* type |
| Genus | *Genisteae* | *Cytisus* type*, Ulex, Ulex* type*, Cytisus/Genista, Cytisus/Genista* type*, Cytisus/Ulex, Genista, Genista* type*, Genista/Ulex, Sarothamnus scoparius, Spartium* |
| Family | Gentianaceae | *Centaurium, Gentiana purpurea* type*,* Gentianaceae*, Gentianella, Gentianella campestris type, Swertia perennis, Centaurium maritimum, Centaurium* type*, Gentiana, Gentiana lutea/Gentiana pneumonanthe, Gentiana nivalis* type*, Gentiana pneumonanthe, Gentiana pneumonanthe* type*, Gentiana purpurea* |
| Family | Geraniaceae | *Erodium,* Geraniaceae*, Geranium, Geranium sylvaticum* type*, Geranium* type |
| Genus | *Halimium* | *Halimium, Halimium* type |
| Genus | *Hedera* | *Hedera, Hedera helix, Hedera* type |
| Genus | *Helianthemum* | *Helianthemum, Helianthemum croceum type, Helianthemum nummularium* type*, Helianthemum salicifolium* type*, Helianthemum* type |
| Genus | *Helleborus* | *Helleborus, Helleborus foetidus, Helleborus viridis* type |
| Genus | *Hippophae* | *Hippophae, Hippophae rhamnoides* |
| Genus | *Huperzia* | *Huperzia selago, Lycopodium selago* |
| Family | Hymenophyllaceae | *Hymenophyllum tunbrigense, Hymenophyllum wilsonii, Trichomanes speciosum* |
| Family | Hypericaceae | *Hypericum, Hypericum hyssopifolium, Hypericum perforatum* type*, Hypericum pulchrum* type*, Hypericum* type |
| Genus | *Ilex* | *Ilex, Ilex aquifolium* |
| Genus | *Impatiens* | *Impatiens* |
| Family | Iridaceae | *Crocus,* Iridaceae*, Iris, Iris pseudacorus, Iris pseudacorus* type*, Iris* type |
| Genus | *Jasminum* | *Jasminum, Jasminum fruticans, Jasminum* type |
| Family | Juglandaceae | Juglandaceae |
| Genus | *Juglans* | *Juglans, Juglans regia* |
| Family | Juncaceae | Juncaceae*, Juncus, Luzula* |
| Genus | *Kalmia* | *Loiseleuria procumbens* |
| Genus | *Koenigia* | *Koenigia islandica* |
| Genus | *Laburnum* | *Laburnum anagyroides* |
| Family | Lamiaceae | *Ajuga, Lycopus* type*, Lycopus/Mentha, Marrubium, Mentha, Mentha* type*, Mentha/Thymus, Origanum vulgare, Phlomis, Prunella, Prunella* type*, Ballota, Prunella vulgaris* type*, Scutellaria, Scutellaria* type*, Sideritis, Stachys, Stachys sylvatica* type*, Stachys sylvestris, Stachys* type*, Thymus, Thymus serpyllum, Galeopsis, Thymus* type*, Galeopsis* type*, Glechoma* type*,* Lamiaceae*, Lamium, Lamium* type*, Lycopus* |
| Genus | *Larix* | *Larix, Larix decidua, Larix decidua* type*, Larix/Pseudotsuga* |
| Genus | *Lavandula* | *Lavandula stoechas* type |
| Genus | *Ledum* | *Ledum, Ledum palustre, Ledum* type |
| Genus | *Ligustrum* | *Ligustrum, Ligustrum vulgare, Ligustrum vulgare* type |
| Family | Liliaceae | *Allium, Lilium martagon* type*, Lloydia serotina, Allium* type*, Anthericum, Anthericum* type*, Fritillaria lusitanica, Fritillaria* type*, Gagea,* Liliaceae*, Lilium* |
| Family | Linaceae | Linaceae*, Radiola linoides* |
| Genus | *Linnaea* | *Linnaea, Linnaea borealis* |
| Genus | *Linum* | *Linum, Linum bienne* type*, Linum catharticum, Linum catharticum* type*, Linum* type |
| Genus | *Lonicera* | *Lonicera, Lonicera caerulea, Lonicera hispida, Lonicera implexa, Lonicera periclymenum, Lonicera periclymenum* type*, Lonicera* type*, Lonicera xylosteum* type |
| Family | Loranthaceae | Loranthaceae |
| Genus | *Lycopodiella* | *Lepidotis inundata, Lycopodium inundatum* |
| Genus | *Lycopodium* | *Diphasiastrum, Lycopodium alpinum, Lycopodium annotinum, Lycopodium annotinum* type*, Lycopodium clavatum, Lycopodium clavatum* type*, Lycopodium complanatum, Lycopodium dubium, Lycopodium* type*, Diphasiastrum alpinum, Diphasium, Diphasium alpinum, Diphasium alpinum* type*, Diphasium complanatum, Diphasium tristachyum, Diphasium* type*, Lycopodium* |
| Genus | *Lysimachia* | *Lysimachia, Lysimachia maritima, Lysimachia nemorum, Lysimachia thyrsiflora, Lysimachia* type*, Lysimachia vulgaris, Lysimachia vulgaris* type |
| Family | Lythraceae | Lythraceae*, Lythrum, Lythrum salicaria, Lythrum salicaria* type*, Lythrum salicaria/Lythrum hyssopifolia, Peplis* |
| Family | Magnoliaceae | Magnoliaceae |
| Genus | *Malus* | *Malus, Malus* type |
| Family | Malvaceae | *Hibiscus, Lavatera* type*, Malva, Malva sylvestris* type*,* Malvaceae*, Sterculia* |
| Family | Melanthiaceae | *Veratrum, Veratrum* type |
| Genus | *Mercurialis* | *Mercurialis, Mercurialis annua, Mercurialis annua* type*, Mercurialis perennis, Mercurialis perennis* type |
| Genus | *Moltkia* | *Moltkia* |
| Family | Montiaceae | *Montia* |
| Family | Moraceae | Moraceae*, Morus, Morus alba, Morus alba* type*, Morus nigra* |
| Genus | *Myrica* | *Corylus/Myrica, Myrica, Myrica gale, Myrica gale* type*, Myrica* type |
| Genus | *Myricaria* | *Myricaria* |
| Family | Myrtaceae | Myrtaceae*, Myrtus, Myrtus communis* |
| Family | Nartheciaceae | *Narthecium, Narthecium ossifragum, Narthecium* type |
| Genus | *Nerium* | *Nerium, Nerium oleander* |
| Genus | *Nigella* | *Nigella* |
| Family | Nitrariaceae | *Nitraria, Peganum harmala* |
| Genus | *Olea* | *Olea, Olea europaea, Olea* type |
| Family | Oleaceae | *Fontanesia/Phillyrea, Olea/Ligustrum,* Oleaceae |
| Family | Onagraceae | *Chamaenerion, Chamaenerion angustifolium, Circaea, Epilobium, Epilobium* type*,* Onagraceae |
| Genus | *Ononis* | *Ononis, Ononis* type |
| Family | Ophioglossaceae | *Botrychium, Botrychium lunaria, Botrychium lunaria* type*, Botrychium* type*,* Ophioglossaceae*, Ophioglossum, Ophioglossum vulgatum* |
| Family | Orchidaceae | *Neottia* type*,* Orchidaceae |
| Family | Orobanchaceae | *Euphrasia, Euphrasia* type*, Melampyrum, Pedicularis, Pedicularis oederi* type*, Pedicularis palustris* type*, Pedicularis* type*, Rhinanthus, Rhinanthus* type |
| Family | Osmundaceae | *Osmunda, Osmunda regalis* |
| Family | Oxalidaceae | Oxalidaceae*, Oxalis, Oxalis acetosella, Oxalis stricta* |
| Genus | *Oxyria/Rumex* | *Oxyria, Rumex acetosa/Rumex acetosella, Rumex acetosa/Rumex acetosella* type*, Rumex acetosa/Rumex scutatus* type*, Rumex acetosella, Rumex acetosella* type*, Rumex alpestris, Rumex alpinus, Rumex alpinus* type*, Rumex aquaticus, Rumex aquaticus* type*, Oxyria digyna, Rumex conglomeratus* type*, Rumex crispus, Rumex crispus* type*, Rumex hydrolapathum, Rumex hydrolapathum* type*, Rumex longifolius, Rumex longifolius* type*, Rumex obtusifolius* type*, Rumex patentia* type*, Rumex sanguineus* type*, Oxyria* type*, Rumex* type*, Oxyria/Rumex, Oxyria/Rumex* type*, Rumex, Rumex acetosa, Rumex acetosa* type*, Rumex acetosa/Oxyria* type |
| Genus | *Paeonia* | *Paeonia* |
| Genus | *Paliurus* | *Paliurus, Paliurus spina-christi* |
| Family | Papaveraceae | *Chelidonium majus, Papaver, Papaver argemone, Papaver rhoeas* type*, Papaver* type*,* Papaveraceae*, Roemeria, Corydalis, Corydalis solida* type*, Corydalis* type*, Fumana, Fumaria, Fumaria officinalis* type*, Glaucium, Hypecoum* |
| Genus | *Parrotia* | *Parrotia persica* |
| Genus | *Periploca* | *Periploca* |
| Genus | *Phillyrea* | *Olea/Phillyrea, Phillyrea, Phillyrea angustifolia, Phillyrea angustifolia* type*, Phillyrea media, Phillyrea* type |
| Family | Phyllanthaceae | *Andrachne, Andrachne telephioides* |
| Genus | *Picea* | *Picea, Picea abies, Picea abies subsp abies* |
| Genus | *Picea orientalis* | *Picea orientalis* |
| Genus | *Pinus* | *Pinus* |
| Genus  sub-group | *Pinus (diploxylon)* | *Pinus (Diploxylon), Pinus subg. Pinus, Pinus sylvestris, Pinus sylvestris* type*, Pinus sylvestris/Pinus nigra* type*, Pinus* type*, Pinus halepensis, Pinus nigra* type*, Pinus non-cembra, Pinus pinaster, Pinus pinaster* type*, Pinus pinaster/Pinus halepensis, Pinus pinea* type*, Pinus pinea/Pinus halepensis* type |
| Genus  sub-group | *Pinus (haploxylon)* | *Pinus (Haploxylon), Pinus cembra, Pinus cembra* type*, Pinus peuce, Pinus sibirica* |
| Genus | *Pistacia* | *Pistacia, Pistacia lentiscus, Pistacia lentiscus* type*, Pistacia terebinthus, Pistacia terebinthus* type*, Pistacia* type |
| Family | Plantaginaceae | *Globularia, Plantago coronopus* type*, Plantago cylindrica* type*, Plantago lanceolata, Plantago lanceolata* type*, Plantago lusitanica, Plantago major, Plantago major* type*, Plantago major/Plantago media, Plantago maririma, Plantago maritima, Gratiola officinalis, Plantago maritima* type*, Plantago media, Plantago media* type*, Plantago media/Plantago major, Plantago media/Plantago major* type*, Plantago montana* type*, Plantago ovata* type*, Plantago psyllium* type*, Plantago tenuiflora* type*, Plantago* type*,* Plantaginaceae*, Plantago, Plantago afra* type*, Plantago albicans, Plantago alpina, Plantago alpina* type*, Plantago coronopus* |
| Genus | *Platanus* | *Platanus, Platanus orientalis, Platanus* type |
| Family | Plumbaginaceae | *Acantholimon, Limonium vulgare,* Plumbaginaceae*, Plumbago, Armeria, Armeria maritima, Armeria maritima subsp elongata, Armeria* type*, Armeria/Limonium, Armeria/Limonium type, Limonium, Limonium* type |
| Family | Poaceae | *Anthoxanthum, Stipa, Deschampsia, Elymus, Lygeum, Lygeum spartum, Nardus, Poa/Festuca* type*,* Poaceae*, Setaria* |
| Family | Polemoniaceae | Polemoniaceae*, Polemonium* |
| Family | Polygalaceae | *Polygala, Polygala* type*, Polygala vulgaris, Polygala vulgaris* type*,* Polygalaceae*, Polygaloides chamaebuxus* |
| Family | Polygonaceae | *Atraphaxis, Persicaria amphibia,* Polygonaceae*, Pteropyrum, Rheum, Rheum ribes, Rheum* type*, Bilderdykia convolvulus, Bilderdykia convolvulus* type*, Bistorta officinalis* type*, Bistorta vivipara, Calligonum, Fallopia convolvulus, Fallopia convolvulus* type*, Persicaria* |
| Genus | *Polygonum* | *Polygonum, Polygonum convolvulus, Polygonum convolvulus* type*, Polygonum oxyspermum* type*, Polygonum persicaria, Polygonum persicaria* type*, Polygonum sect. Persicaria, Polygonum* type*, Polygonum viviparum, Polygonum alpinum, Polygonum amphibium, Polygonum amphibium* type*, Polygonum aviculare, Polygonum aviculare* type*, Polygonum bistorta, Polygonum bistorta* type*, Polygonum bistorta/Polygonum viviparum* |
| Genus | *Polypodiales* | *Aspidium, Blechnum, Blechnum spicant, Cystopteris, Cystopteris fragilis, Cystopteris* type*, Dryopteridaceae/Polypodiaceae, Dryopteris, Dryopteris carthusiana, Dryopteris carthusiana* type*, Dryopteris cristata, Asplenium, Dryopteris cristata* type*, Dryopteris dilatata, Dryopteris dilatata* type*, Dryopteris filix-mas, Dryopteris filix-mas* type*, Dryopteris* type*, Dryopteris/Thelypteris, Grammitis, Gymnocarpium, Gymnocarpium dryopteris, Asplenium nidus, Pilularia,* Polypodiaceae*,* Polypodiales*, Polypodium, Polypodium vulgare, Polypodium vulgare* type*, Polystichum, Polystichum* type*, Thelypteris, Thelypteris palustris, Asplenium* type*, Thelypteris palustris* type*, Thelypteris phegopteris, Thelypteris* type*, Asplenium viride, Athyrium, Athyrium alpestre* type*, Athyrium distentifolium* type*, Athyrium filix-femina* |
| Genus | *Populus* | *Populus, Populus tremula, Populus tremula* type |
| Family | Portulacaceae | Portulacaceae |
| Genus | *Potentilla* | *Potentilla, Potentilla aurea, Potentilla micrantha* type*, Potentilla recta, Potentilla* type |
| Family | Primulaceae | *Anagallis, Primula, Primula clusiana* type*, Primula farinosa, Primula farinosa* type*, Primula hirsuta* type*, Primula* type*, Primula veris* type*, Primula vulgaris* type*,* Primulaceae*, Soldanella, Anagallis arvensis, Trientalis, Trientalis europaea, Anagallis arvensis* type*, Anagallis tenella, Anagallis* type*, Androsace, Androsaceae, Cyclamen, Cyclamen hederifolium* |
| Genus | *Prosopis* | *Lagonychium* type*, Prosopis* |
| Genus | *Prunus* | *Prunus, Prunus avium, Prunus padus, Prunus spinosa* type*, Prunus* type |
| Family | Pteridaceae | *Adiantum, Cheilanthes, Cryptogramma, Cryptogramma crispa, Cryptogramma crispa* type*, Pteris* |
| Genus | *Pterocarya* | *Pterocarya, Pterocarya fraxinifolia* |
| Genus | *Punica* | *Punica* |
| Genus | *Pyrus* | *Pyrus, Pyrus* type |
| Genus | *Quercus* (deciduous) | *Quercus, Quercus robur* type*, Quercus robur/Quercus petraea, Quercus (deciduous), Quercus cerris, Quercus cerris* type*, Quercus deciduous, Quercus ithaburensis, Quercus ithaburensis* type*, Quercus petraea, Quercus robur* |
| Genus | *Quercus* (evergreen) | *Quercus (evergreen), Quercus rotundifolia* type*, Quercus suber, Quercus suber* type*, Quercus calliprinos, Quercus calliprinos* type*, Quercus coccifera, Quercus coccifera* type*, Quercus coccifera/Quercus ilex, Quercus evergreen, Quercus ilex, Quercus ilex* type |
| Genus | *Quercus* (intermediate) | *Quercus canariensis type, Quercus cerris/Quercus suber, Quercus cerris/Quercus suber* type*, Quercus faginea, Quercus faginea/Quercus pubescens, Quercus faginea/Quercus pyrenaica, Quercus pubescens* type*, Quercus pyrenaica* type*, Quercus robur/Quercus pubescens* type |
| Family | Ranunculaceae | *Anemone, Anemone nemorosa, Anemone nemorosa type, Anemone nemorosa* type*/Hepatica nobilis, Anemone nemorosa/Anemone ranunculoides, Anemone* type*, Hepatica, Pulsatilla,* Ranunculaceae |
| Genus | *Ranunculus* | *Ranunculus, Ranunculus ficaria* type*, Ranunculus flammula* type*, Ranunculus glacialis* type*, Ranunculus montanus* type*, Ranunculus muricatus* type*, Ranunculus nivalis* type*, Ranunculus parviflorus, Ranunculus repens* type*, Ranunculus sceleratus* type*, Ranunculus* type*, Ranunculus acer* type*, Ranunculus aconitifolius, Ranunculus aconitifolius* type*, Ranunculus acris, Ranunculus acris* type*, Ranunculus arvensis, Ranunculus arvensis* type*, Ranunculus asiaticus* type |
| Family | Resedaceae | *Reseda, Reseda lutea* type*,* Resedaceae |
| Family | Rhamnaceae | Rhamnaceae |
| Genus | *Rhamnus* | *Rhamnus, Rhamnus catharticus, Rhamnus* type |
| Genus | *Rhododendron* | *Rhododendron, Rhododendron ferrugineum, Rhododendron* type |
| Genus | *Rhus* | *Rhus, Rhus coriaria* |
| Genus | *Ribes* | *Ribes, Ribes* type |
| Family | Rosaceae | *Agrimonia, Fragaria* type*, Geum, Geum rivale* type*, Geum* type*, Pirus* type*, Prunus/Rubus* type*, Pyrus/Malus, Rosa, Rosa canina* type*, Rosa* type*, Agrimonia eupatoria, Rosa/Prunus,* Rosaceae*, Spiraea, Alchemilla, Alchemilla* type*, Filipendula, Filipendula* type*, Filipendula ulmaria, Filipendula vulgaris, Fragaria* |
| Genus | *Rosmarinus* | *Rosmarinus, Rosmarinus* type |
| Family | Rubiaceae | *Asperula, Galium, Galium* type*,* Rubiaceae*, Theligonum* |
| Genus | *Rubus* | *Rubus, Rubus arcticus, Rubus arcticus* type*, Rubus chamaemorus, Rubus fruticosus* type*, Rubus idaeus* type*, Rubus saxatilis, Rubus* type |
| Genus | *Ruscus* | *Ruscus* |
| Family | Rutaceae | *Haplophyllum, Ruta,* Rutaceae |
| Genus | *Salix* | *Salix, Salix glauca* type*, Salix helvetica* type*, Salix herbacea, Salix herbacea* type*, Salix herbacea/Salix reticulata, Salix pentandra* type |
| Genus | *Salvia* | *Salvia, Salvia verticillata* type |
| Genus | *Sambucus* | *Sambucus, Sambucus ebulus, Sambucus nigra, Sambucus nigra* type*, Sambucus nigra/Sambucus racemosa, Sambucus racemosa, Sambucus* type |
| Genus | *Sanguisorba group* | *Poterium, Sanguisorba, Sanguisorba minor, Sanguisorba minor ssp minor, Sanguisorba minor* type*, Sanguisorba officinalis, Sanguisorba* type*, Sarcopoterium* |
| Family | Santalaceae | *Arceuthobium oxycedri, Comandra elegans, Osyris alba* type*, Thesium* |
| Family | Saxifragaceae | *Chrysosplenium, Saxifraga oppositifolia, Saxifraga oppositifolia* type*, Saxifraga paniculata* type*, Saxifraga stellaris, Saxifraga stellaris* type*, Saxifraga tricuspidata,* Saxifragaceae*, Chrysosplenium* type*, Micranthes nivalis* type*, Saxifraga, Saxifraga cernua* type*, Saxifraga cespitosa* type*, Saxifraga foliolosa* type*, Saxifraga granulata, Saxifraga granulata* type |
| Family | Scrophulariaceae | *Antirrhinum* type*, Scrophularia/Verbascum,* Scrophulariaceae*, Verbascum, Verbascum* type*, Veronica, Veronica* type*, Antirrhinum/Linaria, Digitalis, Digitalis purpurea* type*, Digitalis* type*, Linaria, Linaria* type*, Scrophularia, Scrophularia* type |
| Genus | *Smilax* | *Smilax* |
| Family | Solanaceae | *Capsicum* type*, Lycium,* Solanaceae*, Solanum, Solanum dulcamara, Solanum nigrum, Solanum nigrum* type |
| Genus | *Sorbus* | *Sorbus, Sorbus aria, Sorbus aucuparia, Sorbus aucuparia* type*, Sorbus* type |
| Genus | *Styrax* | *Styrax officinalis* |
| Genus | *Suaeda* | *Suaeda, Suaeda* type |
| Genus | *Syringa* | *Syringa* |
| Genus | *Tamarix* | *Tamarix* |
| Genus | *Taxus* | *Taxus, Taxus baccata* |
| Genus | *Teucrium* | *Teucrium, Teucrium* type |
| Genus | *Thalictrum* | *Thalictrum, Thalictrum alpinum, Thalictrum aquilegiifolium, Thalictrum flavum* type*, Thalictrum lucidum, Thalictrum* type |
| Family | Thymelaeaceae | *Thymelaea,* Thymelaeaceae |
| Genus | *Tilia* | *Tilia, Tilia cordata, Tilia cordata* type*, Tilia platyphyllos, Tilia platyphyllos* type |
| Genus | *Tofieldia* | *Tofieldia* |
| Genus | *Trollius* | *Trollius, Trollius europaeus, Trollius* type |
| Genus | *Ulmus* | *Ulmus, Ulmus glabra, Ulmus glabra* type*, Ulmus minor* |
| Genus | *Ulmus/Zelkova* | *Ulmus/Zelkova, Zelkova, Zelkova* type |
| Family | Urticaceae | *Parietaria, Parietaria/Urtica, Urtica, Urtica dioica, Urtica dioica* type*, Urtica pilulifera* type*, Urtica* type*, Urtica urens,* Urticaceae |
| Genus | *Vaccinium* | *Vaccinium, Vaccinium myrtillus, Vaccinium myrtillus type, Vaccinium oxycoccos, Vaccinium* type*, Vaccinium uliginosum* type*, Vaccinium/Oxycoccus* |
| Family | Valerianaceae | *Centranthus, Valerianella, Valeriana, Valeriana dioica* type*, Valeriana officinalis, Valeriana officinalis* type*, Valeriana sambucifolia* type*, Valeriana tripteris* type*, Valeriana* type*,* Valerianaceae |
| Family | Verbenaceae | *Verbena, Verbena officinalis* |
| Genus | *Viburnum* | *Viburnum, Viburnum opulus, Viburnum opulus* type*, Viburnum* type |
| Family | Violaceae | *Viola, Viola arvensis* type*, Viola canina* type*, Viola palustris, Viola palustris* type*, Viola tricolor, Viola tricolor/Viola arvensis,* Violaceae |
| Genus | *Viscum* | *Viscum, Viscum album, Viscum album* type*, Viscum* type |
| Genus | *Vitex* | *Vitex* |
| Genus | *Ziziphus* | *Ziziphus* type*, Zizyphus, Zizyphus lotus* |
| Family | Zygophyllaceae | *Tribulus, Tribulus terrestris,* Zygophyllaceae*, Zygophyllum* |

Supplementary Table 2: Comparison of the quality of reconstructions based on training datasets constructed in different ways or the modern data set for the whole of the SPECIAL Modern Pollen Data Set (SMPDS) and for the data set encompassing only the Eastern Mediterranean-Black Sea Caspian Corridor (EMBSeCBIO) region. Assessments are made on the accuracy with respect to only the dominant biome and to the dominant and sub-dominant biomes identified in a 20 x 20 km^2^ search window around each sampling point according to the Potential Natural Vegetation (PNV) Map of Hengl et al. (2018). We give both the accuracy and the balanced accuracy metrics for each split of the training and testing datasets.

|  |  | **SMPDS region** | | | | **EMBSeCBIO region** | | | | |
| --- | --- | --- | --- | --- | --- | --- | --- | --- | --- | --- |
|  |  | **Dominant biome** | | **Dominant and sub-dominant** | | **Dominant biome** | | **Dominant and sub-dominant** | |  |
| **Training dataset** | **Testing dataset** | **Accuracy** | **Balanced accuracy** | **Accuracy** | **Balanced accuracy** | **Accuracy** | **Balanced accuracy** | **Accuracy** | **Balanced accuracy** |  |
| Random selection of 70% of the data | Random selection of 30% of the data | 67.30 | 62.07 | 78.89 | 77.78 | 65.36 | 58.76 | 79.45 | 75.64 |  |
| Random selection with 0% from SMPDS, 50% from EMBSeCBIO. | Random selection of combined data set to produce test data sets the same size as the training set | 63.93 | 57.00 | 75.63 | 71.49 | 71.18 | 67.03 | 82.10 | 78.46 |  |
| Random selection with 50% of the samples for each biome from SMPDS and 50% from EMBSeCBIO; each class limited to number of samples of the median size class. | Random selection of samples of the same size as the training set | 64.66 | 63.55 | 75.73 | 78.00 | 68.74 | 64.40 | 79.45 | 77.10 |  |
| Optimum sampling of bioclimatic space of each class to have a maximum of 1000 samples the training dataset | All the samples not used for the training dataset | 63.86 | 62.80 | 75.18 | 78.34 | 64.70 | 56.92 | 77.70 | 72.01 |  |
| Random down-sampling of classes towards the median size class (GRAM). 70% of down-sampled data used for training | 30% of down-sampled data for testing | 65.75 | 65.88 | 75.68 | 75.58 | 62.30 | 64.22 | 74.21 | 77.34 |  |
| Samples from large lakes removed; random down-sampling of classes towards the median size class (GRAM); 70% of down-sampled data used for training. | 30% of down-sampled data used for testing | 66.32 | 66.49 | 77.49 | 76.52 | 63.82 | 64.54 | 75.42 | 78.32 |  |

Supplementary Table 3: Comparison of the quality of reconstructions based on using different areas around each sample to determine the observed vegetation type and on using different training and testing data partitioning ratios. The size of the search window is given in km. For the ratio of the size of the training and testing data sets, a value of 70:30 means that 70% of the data are used as the training set and 30% of the data are used as the test set. Assessments are made on the accuracy with respect to only the dominant biome and to the dominant and sub-dominant biomes identified in each search window around each sampling point according to the Potential Natural Vegetation (PNV) Map of Hengl et al. (2018) for the modern data set for the whole of the SPECIAL Modern Pollen Data Set (SMPDS) and for the data set encompassing only the Eastern Mediterranean-Black Sea Caspian Corridor (EMBSeCBIO) region. We give both the accuracy and the balanced accuracy metrics for each assessment.

|  |  | **SMPDS REGION** | | | | **EMBSECBIO REGION** | | | |
| --- | --- | --- | --- | --- | --- | --- | --- | --- | --- |
|  |  | **Dominant** | | **Dominant and subdominant** | | **Dominant** | | **Dominant and subdominant** | |
| **Size of the search window (km)** | **Training - testing data partitioning ratio** | **Accuracy** | **Balanced accuracy** | **Accuracy** | **Balanced accuracy** | **Accuracy** | **Balanced accuracy** | **Accuracy** | **Balanced accuracy** |
| 12x12 | 50:50 | 58.76 | 58.80 | 73.71 | 73.23 | 62.25 | 57.23 | 77.79 | 75.84 |
| 12x12 | 60:40 | 62.00 | 63.18 | 73.50 | 74.22 | 62.32 | 57.81 | 76.45 | 74.05 |
| 12x12 | 70:30 | 63.09 | 63.41 | 75.17 | 75.53 | 64.18 | 66.15 | 77.88 | 80.03 |
| 12x12 | 75:25 | 62.35 | 62.55 | 72.06 | 72.31 | 62.91 | 62.66 | 76.04 | 74.96 |
| 12x12 | 80:20 | 59.18 | 59.59 | 73.98 | 74.45 | 62.47 | 60.19 | 76.04 | 74.07 |
| 20x20 | 50:50 | 61.76 | 62.19 | 74.03 | 74.55 | 62.73 | 60.07 | 76.28 | 78.22 |
| 20x20 | 60:40 | 61.24 | 61.52 | 73.90 | 73.93 | 61.20 | 57.90 | 74.75 | 73.94 |
| 20x20 | 70:30 | 65.75 | 65.88 | 75.68 | 75.58 | 62.30 | 64.22 | 74.21 | 77.34 |
| 20x20 | 75:25 | 64.58 | 64.75 | 76.25 | 76.49 | 64.48 | 59.51 | 76.50 | 75.70 |
| 20x20 | 80:20 | 68.37 | 69.23 | 75.00 | 75.36 | 63.72 | 59.42 | 76.72 | 75.95 |
| 25x25 | 50:50 | 60.34 | 60.85 | 73.21 | 73.43 | 63.48 | 54.85 | 74.81 | 71.72 |
| 25x25 | 60:40 | 60.85 | 61.42 | 73.54 | 73.76 | 66.04 | 63.39 | 76.92 | 75.90 |
| 25x25 | 70:30 | 60.42 | 61.07 | 74.20 | 74.33 | 63.96 | 58.03 | 76.04 | 75.44 |
| 25x25 | 75:25 | 61.92 | 62.58 | 71.13 | 71.45 | 65.82 | 58.88 | 78.24 | 75.08 |
| 25x25 | 80:20 | 66.32 | 66.38 | 74.21 | 74.91 | 66.37 | 59.28 | 78.57 | 76.10 |
| 40x40 | 50:50 | 63.29 | 63.76 | 77.03 | 76.74 | 59.45 | 54.70 | 73.19 | 76.14 |
| 40x40 | 60:40 | 65.27 | 65.26 | 76.75 | 76.26 | 61.98 | 55.93 | 76.48 | 77.19 |
| 40x40 | 70:30 | 67.41 | 67.69 | 76.30 | 76.31 | 62.57 | 54.29 | 75.19 | 74.56 |
| 40x40 | 75:25 | 68.58 | 68.93 | 76.55 | 77.15 | 64.65 | 56.08 | 79.14 | 77.45 |
| 40x40 | 80:20 | 67.96 | 68.07 | 80.11 | 80.30 | 64.76 | 57.59 | 77.50 | 76.29 |
| 50x50 | 50:50 | 65.27 | 65.92 | 76.36 | 76.28 | 61.43 | 61.16 | 75.52 | 76.00 |
| 50x50 | 60:40 | 68.07 | 65.31 | 82.06 | 82.26 | 65.38 | 63.54 | 80.36 | 78.44 |
| 50x50 | 70:30 | 67.13 | 65.61 | 77.97 | 77.99 | 66.93 | 64.12 | 80.50 | 79.10 |
| 50x50 | 75:25 | 64.98 | 65.67 | 78.06 | 79.04 | 66.25 | 65.29 | 79.13 | 78.97 |
| 50x50 | 80:20 | 65.63 | 66.12 | 79.17 | 78.73 | 67.54 | 64.47 | 80.70 | 79.69 |

Supplementary Table 4. Comparison of the quality of reconstructions for the modern data set for the whole of the SPECIAL Modern Pollen Data Set (SMPDS) and for the data set encompassing only the Eastern Mediterranean-Black Sea Caspian Corridor (EMBSeCBIO) region based on using different values for ε in equation 1 (range from 0.01 to 1). Assessments are made on the accuracy with respect to only the dominant biome and to the dominant and sub-dominant biomes identified in a 20 x 20 km^2^ search window around each sampling point according to the Potential Natural Vegetation (PNV) Map of Hengl et al. (2018). We give both the accuracy and the balanced accuracy metrics for assessment.

|  | **SMPDS region** | | | | **EMBSeCBIO region** | | | |
| --- | --- | --- | --- | --- | --- | --- | --- | --- |
|  | **Dominant biome** | | **Dominant and Subdominant** | | **Dominant biome** | | **Dominant and Subdominant** | |
| **Value for ε** | **Accuracy** | **Balanced accuracy** | **Accuracy** | **Balanced accuracy** | **Accuracy** | **Balanced accuracy** | **Accuracy** | **Balanced accuracy** |
| 0.01 | 66.10 | 65.93 | 73.63 | 72.59 | 66.92 | 60.06 | 78.86 | 77.16 |
| 0.05 | 63.70 | 63.64 | 71.92 | 71.15 | 66.01 | 62.23 | 77.38 | 77.80 |
| 0.1 | 64.04 | 64.17 | 73.29 | 73.07 | 65.03 | 63.03 | 77.16 | 79.58 |
| 0.5 | 65.75 | 65.88 | 75.68 | 75.58 | 62.30 | 64.22 | 74.21 | 77.34 |
| 0.8 | 66.78 | 66.86 | 75.68 | 75.60 | 62.19 | 63.68 | 74.43 | 78.25 |
| 1 | 66.78 | 66.86 | 75.68 | 75.67 | 61.60 | 62.99 | 74.18 | 77.69 |

Supplementary Table 5: Plant functional types (PFTs) included in each biome in the biomisation procedure. The allocations of PFTs to biomes follows Marinova et al. (2018), but has been modified to take account of the amalgamation of some biomes in our analyses.

| **Biomes** | **Biomes in Marinova et al. (2017)** | **Constituent plant functional types** |
| --- | --- | --- |
| TUND | Tundra (TUND) | Arctic forb, sedge graminoid, arctic dwarf shrub, arctic low-to-high shrub |
| DESE | Desert (DESE) | Halophyte, rosette or cushion forb, succulent, switch plant, tuft tree |
| GRAM | Graminoids with forbs (GRAM) | Grass graminoid, geophyte, other forb |
| XSHB | Xeric shrubland (XSHB) | Drought-tolerant forb, switch plant, xerophytic shrub |
| WTSFS | Warm-temperate evergreen sclerophyll broadleaf shrubland (WTSHB), Warm-temperate deciduous malacophyll broadleaf forest (WTDF), Warm-temperate evergreen needleleaf and sclerophyll broadleaf forest (WTEF) | Warm-temperate low-to-high shrub, temperate low-to-high shrub, warm-temperate sclerophyll tree, temperate (spring frost tolerant) cold-deciduous malacophyll broadleaved tree temperate (spring frost intolerant) cold-deciduous malacophyll broadleaved tree, climber/liana/vine, warm-temperate needle-leaved evergreen tree, warm-temperate evergreen malacophyll broadleaved tree, eurythermic evergreen needle-leaved tree |
| CENF | Cold evergreen needleleaf forest (CENF), Cool evergreen needleleaf forest (COOL) | Boreal low-to-high shrub, boreal cold-deciduous malacophyll broadleaved tree, boreal evergreen needle-leaved tree, boreal needle-leaved deciduous tree, eurythermic evergreen needle-leaved tree, Boreal cold-deciduous malacophyll broadleaved tree, cool-temperate evergreen needle-leaved tree, temperate (spring frost tolerant) cold-deciduous malacophyll broadleaved tree, temperate evergreen needle-leaved tree |
| TEDE | Temperate deciduous malacophyll broadleaf forest (TEDE) | Temperate (frost-induced late budburst) cold-deciduous malacophyll broadleaved tree, temperate (spring frost intolerant) cold-deciduous malacophyll broadleaved tree, temperate (spring frost tolerant) cold-deciduous malacophyll broadleaved tree, eurythermic evergreen needle-leaved tree, climber/liana/vine |
| CMIX | Cool mixed evergreen needleleaf and deciduous broadleaf forest (CMIX) | Cool-temperate evergreen needle-leaved tree, eurythermic evergreen needle-leaved tree, temperate (frost-induced late budburst) cold-deciduous malacophyll broadleaved tree, temperate (spring frost tolerant) cold-deciduous malacophyll broadleaved tree, temperate evergreen needle-leaved tree |
| ENWD | Evergreen needleleaf woodland (ENWD), Deciduous broadleaf woodland (DBWD) | Warm-temperate low-to-high shrub, other forb, eurythermic evergreen needle-leaved tree, warm-temperate needle-leaved evergreen tree, warm-temperate evergreen malacophyll broadleaved tree, temperate (spring frost intolerant) cold-deciduous malacophyll broadleaved tree, temperate low-to-high shrub |

Supplementary Table 6: Taxon allocation to plant functional types (PFTs) used in the biomisation reconstructions. The allocations of taxa to PFTs es follows Marinova et al. (2018) but has been modified to take account of the amalgamation of some biomes in our analyses.

| **Plant functional type** | **Constituent taxa** |
| --- | --- |
| Arctic forb | *Aconitum*, *Aconitum* type, Androsace, *Anemone*, *Anemone nemorosa* type, *Anemone* type, *Aquilegia* type,Campanulaceae, *Cardamine*, *Drosera*, *Gentiana*, *Gentiana nivalis* type, *Gentiana pneumonanthe* type, Gentianaceae, *Gentianella campestris* type, Herbs, *Jasione*, *Parnassia*, *Parnassia palustris*, *Phyteuma*, *Phyteuma* type, *Pinguicula*, Polygonaceae, *Polygonum*, *Polygonum* type, *Pulsatilla*, Ranunculaceae, *Rosa*, *Rosa* type, *Sagina*, *Saussurea*, *Saxifraga*, *Saxifraga hirsuta* type, *Saxifraga nivalis* type, *Saxifraga oppositifolia*, *Saxifraga oppositifolia* type, *Saxifraga rosacea*, *Saxifraga stellaris* type, Saxifragaceae, Scrophulariaceae, *Thalictrum*, *Thalictrum aquilegifolium*, *Trollius*, *Valeriana*, Valerianaceae, *Veratrum* type |
| Rosette or cushion forb | *Artemisia*, *Artemisia herba-alba* type, *Artemisia* type, Asteraceae, Asteraceae (Liguliflorae), Asteraceae (Tubuliflorae), *Astragalus*, *Astragalus* type, Crassulaceae, *Euphorbia*, *Gundelia* type, Herbs, *Marrubium*, *Phlomis*, *Scabiosa*, *Scabiosa columbaria* type, *Scleranthus*, *Scleranthus* type, *Tribulus*, *Zygophyllum* |
| Drought-tolerant forb | *Achillea*, *Achillea* type, *Adonis* , *Adonis aestivalis* type, *Adonis* type, Amaranthaceae/Chenopodiaceae, *Ambrosia*, *Ambrosia* type, *Armeria*, *Armeria*/*Limonium*, *Artemisia*, *Artemisia* type, *Artemisia vulgaris* type, *Aster*, *Aster* type, *Aster*/*Achillea*, *Aster*/*Achillea* type, Asteraceae, Asteraceae (Liguliflorae), Asteraceae (Tubuliflorae), *Astragalus*, *Astragalus* type, *Astrantia* type, *Atriplex*, Cannabaceae, *Cannabis*, *Cannabis* *sativa*, *Carduus*, *Carduus* type, *Carthamus*, Caryophyllaceae, *Centaurea*, *Centaurea cyanus*, *Centaurea cyanus* type, *Centaurea depressa*, *Centaurea depressa* type, *Centaurea jacea*, *Centaurea jacea* type, *Centaurea nigra* type, *Centaurea scabiosa*, *Centaurea scabiosa* type, *Centaurea solstitialis* type, Chenopodiaceae, Dipsacaceae, *Dipsacus*, *Dipsacus* type, *Echinops*, *Eryngium* type, *Euphorbia*, *Fagopyrum*, *Fagopyrum esculentum*, *Fagopyrum tataricum*, *Glaucium*, *Gundelia* type, *Gypsophila*, *Gypsophila* type, *Helichrysum*, *Heliotropium* type, Herbs, *Herniaria* type, *Hornungia* type, *Jurinea*, *Jurinea* type, *Knautia*, *Knautia arvensis*, *Limonium*, *Noaea* type, *Salvia*, *Scabiosa*, *Scabiosa columbaria* type, *Scabiosa rotata* type, Scrophulariaceae, *Serratula*, *Seseli libanotis* type, *Sideritis*, *Succisa*, *Thymus*, *Verbascum*, *Verbascum* type |
| Other forb | *Acanthus*, *Achillea*, *Achillea* type, *Aconitum*, *Aconitum* type, *Adoxa* type, *Agrimonia*, *Agrimonia eupatoria*, *Agrostemma* type, *Alchemilla*, Amaranthaceae/Chenopodiaceae, *Ambrosia*, *Ambrosia* type, *Ammi* type, *Anagallis*, *Anemone*, *Anemone* type, *Anthemis* type, *Anthriscus* type, Apiaceae, *Apium* type, *Arctium*, *Arctium*/*Jurinea*, *Asperula* type, *Aster*, *Aster* type, *Aster*/*Achillea*, *Aster*/*Achillea* type, Asteraceae, Asteraceae (Liguliflorae), Asteraceae(Tubuliflorae), *Bellis* type, *Beta*, *Bidens* type, Boraginaceae, *Brassica* type, Brassicaceae, Brassicaceae type, *Bunium* type, *Bupleurum*, *Bupleurum* type, *Caltha*, *Caltha* type, *Campanula*, *Campanula* type, Campanulaceae, Cannabaceae, *Capsella* type, *Carduus*, *Carduus* type, Caryophyllaceae, *Centaurea*, *Centranthus*, *Cerastium* type, *Chaerophyllum* type, *Cheilanthes*, *Chelidonium*, Chenopodiaceae, *Chrysosplenium*, *Chrysosplenium* type, Cichoriaceae, *Circaea*, *Cirsium*, *Cirsium* type, *Cirsium*/*Carduus*, *Cirsium*/*Gundelia*, *Conium maculatum*, *Consolida*, Convolvulaceae, *Convolvulus*, *Daucus* type, *Delphinium*, *Delphinium* type, *Dianthus*, *Dianthus* type, *Digitalis*, *Digitalis purpurea* type, *Diphasium alpinum* type, Dipsacaceae, *Dipsacus*, *Dipsacus* type, *Echium*, *Echium* type, *Echium violaceum*, *Epilobium*, *Epilobium* type, *Erodium*, *Euphrasia*, *Falcaria* type, *Ferula*, *Ferula* type, *Filago* type, *Filifolium sibiricum*, *Filipendula*, *Flammula* type, *Fragaria* type, *Fumaria*, *Galium*, *Galium* type, Geraniaceae, *Geranium*, *Geum*, *Geum* type, *Gnaphalium*, *Hedysarum* type, *Helleborus*, *Heracleum*, *Heracleum* type, *Hippocrepis* type, *Hyoscyamus*, *Hypericum*, *Hypericum assyriacum* type, *Hypericum hyssopifolium*, *Hypericum perforatum* type, *Hypericum* type, *Impatiens*, *Lactuca*, *Lathyrus*, *Lathyrus* type, *Legousia*, Leguminosae, *Lepidium*, *Lepidium* type, *Linaceae*, *Linaria*, *Linum*, *Linum* type, *Lithospermum*, *Lotus* type, *Lychnis* type, *Lysimachia*, *Malabaila*, *Malabaila* type, *Malva*, Malvaceae, *Matricaria* type, *Matthiola*, *Medicago*, *Melampyrum*, *Mercurialis*, *Mercurialis annua*, *Mercurialis perennis*, *Myosotis*, *Myosotis* type, *Nigella*, Onagraceae, *Onobrychis*, *Onobrychis* type, *Onosma*, *Origanum vulgare*, *Oxalis*, *Oxyria*, *Oxyria*/*Rumex*, *Papaver*, *Papaver rhoeas* type, Papaveraceae, *Parietaria*, *Paronychia*, *Paronychia*/*Polycnemum*, *Peucedanum* type, *Pimpinella*, *Pimpinella anisum* type, *Pimpinella major* type, *Pimpinella* type, Plantaginaceae, *Plantago*, *Plantago coronopus*, *Plantago coronopus* type, *Plantago lanceolata*, *Plantago lanceolata* type, *Plantago major*, *Plantago major* type, *Plantago major*/*Plantago media*, *Plantago maritima*, *Plantago maritima* type, *Plantago media*, *Plantago media* type, *Plantago ovate*, *Plantago ovata* type, Plumbaginaceae, Polemoniaceae, *Polemonium*, *Polygala*, Polygonaceae, *Polygonum*, *Polygonum* type, Portulacaceae, *Potentilla*, *Potentilla* type, *Primula*, Primulaceae, *Prunella* type, *Pulmonaria* type, Ranunculaceae, *Reseda*, Resedaceae, *Rhinanthus*, *Rhinanthus* type, *Rumex*, *Rumex acetosa*, *Rumex acetosa* type, *Rumex acetosa*/*Rumex acetosella*, *Rumex acetosella*, *Rumex acetosella* type, *Rumex cyprius*, *Rumex hydrolapathum*, *Rumex hydrolapathum* type, *Rumex patentia*, *Rumex patentia* type, *Rumex scutatus* type, *Rumex* type, *Sagina*, *Salvia*, *Sanguisorba*, *Sanguisorba minor*, *Sanguisorba minor* type, *Sanguisorba officinalis*, *Sanguisorba* type, *Sanicula* type, Scrophulariaceae, *Scutellaria*, *Senecio*, *Senecio* type, *Silene*, *Silene dioica* type, *Silene* type, *Silene vulgaris* type, *Sinapis* type, Solanaceae, *Solanum*, *Solanum nigrum*, *Spergula*, *Spergula arvensis* , *Spergula* type, *Spergula*/*Spergularia*, *Spergularia* type, *Stachys*, *Stachys* type, *Stellaria*, *Symphytum*, *Symphytum* type, *Taraxacum*, *Taraxacum* type, *Teucrium*, *Thesium*, *Torilis arvensis* type, *Torilis japonica* type, *Trifolium*, *Trifolium alpestre* type, *Trifolium pratense*, *Trifolium pratense* type, *Trifolium* type, *Turgenia* type, *Urtica*, *Urtica dioica*, *Urtica dioica* type, *Urtica pilulifera* type, *Urtica* type, Urticaceae, *Vaccaria* type, *Valeriana*, Valerianaceae, *Verbascum*, *Verbascum* type, *Verbena*, *Veronica* type, *Vicia*, *Vicia* type, *Viola*, Violaceae, *Xanthium* |
| Halophyte | Amaranthaceae/Chenopodiaceae, *Atriplex*, *Calligonum*, *Ceratoides*, Chenopodiaceae, *Crambe*, *Frankenia*, *Frankenia* hirsute, *Halogeton*, *Halothamnus* type, *Hammada* type, *Lycium*, *Nitraria*, *Peganum*, *Peganum* *harmala*, *Salsola*, *Salsola* type, *Suaeda*, *Suaeda* type, *Tamarix* |
| Geophyte | *Allium*, *Allium* type, *Anthericum* type, Araceae, *Asparagus* type, *Asphodeline*, *Asphodelus*, *Calamus*, *Colchicum*, *Cyclamen*, *Eremurus*, *Fritillaria* type, *Iridaceae*, *Iris*, Liliaceae, *Lilium*, *Maianthemum* type, *Muscari*, *Narthecium* type, *Ornithogalum* type, *Scilla* type, *Scorzonera*, *Scorzonera humilis* type, *Scorzonera* type, *Tulipa sylvestris* type, *Tulipa systola* type |
| Succulent | *Aellenia* type, Amaranthaceae/Chenopodiaceae, Chenopodiaceae, Crassulaceae, *Euphorbia*, *Sedum*, *Sedum* type, Zygophyllum |
| Grass graminoid | *Glyceria* type, *Lygeum*, Poaceae, *Secale*, *Secale* type, *Stipa* |
| Sedge graminoid | *Carex*, *Carex* type, *Cladium*, *Cladium mariscus*, Cyperaceae, *Cyperus*, *Fimbristylis*, Juncaceae, *Juncus*/*Luzula*, *Rhynchospora* type, *Scheuchzeria palustris*, *Schoenoplectus* |
| Arctic dwarf shrub | *Betula*, *Bruckenthalia*, *Dryas* type, Ericaceae, Ericaceae type, *Potentilla*, *Potentilla* type, *Primula*, Primulaceae, *Rheum*, *Rheum* type, *Rubus arcticus*, *Rubus chamaemorus*, *Salix*, *Vaccinium*, *Vaccinium* type, *Vaccinium uliginosum* type, *Veratrum* type |
| Switch plants | *Ephedra*, *Ephedra alata* type, *Ephedra distachya*, *Ephedra distachya* type, *Ephedra fragilis*, *Ephedra fragilis* type, *Ephedra fragilis* var *campylopoda*, *Ephedra major type* |
| Climber/liana/vine | *Calystegia*, *Calystegia sepium*, *Clematis*, *Clematis* type, Convolvulaceae, *Convolvulus*, *Convovulus arvensis*, *Cuscuta*, *Glycine*, *Hedera*, *Hedera helix*, *Humulus*, *Humulus lupulus*, *Lonicera*, *Periploca*, Ranunculaceae, *Smilax*, Solanaceae, *Solanum*, *Solanum dulcamara*, *Tamus communis*, *Vitis*, *Vitis vinifera* |
| Boreal low-to-high shrub | Cotoneaster, *Erica*, *Erica* type, Ericaceae, Ericaceae type, *Myrica*, Pinaceae, *Pinus*, *Pinus* (Diploxylon), *Pinus* subg. *Pinus*, *Ribes*, *Ribes* cf. *montigenum*, *Vaccinium*, *Vaccinium* type |
| Temperate low-to-high shrub | Amaranthaceae/Chenopodiaceae, *Atropa*, Berberidaceae, *Berberis*, *Calluna*, *Calluna vulgaris* type, Chenopodiaceae, Cistaceae, *Cistus*, Convolvulaceae, *Cornus*, *Cornus mas*, *Cornus mas*/*Cornus suecica*, *Cornus sanguinea*, *Cotoneaster*, *Crataegus*, *Crataegus* type, *Daphne*, *Erica*, *Erica* type, Ericaceae, Ericaceae type, *Hippophae*, *Hippophae rhamnoides*, *Lycium*, *Prunus*, *Prunus spinosa* type, *Prunus* type, Rhamnaceae, *Rhamnus*, *Rhododendron*, *Rhododendron ponticum*, *Ribes*, *Rosa*, *Rosa* type, *Rubus fruticosus*, Rutaceae, *Sambucus*, *Sambucus ebulus*, *Sambucus nigra* type, *Sambucus* type, Scrophulariaceae, Thymelaeaceae, *Thymus*, *Viburnum*, *Viburnum* type |
| Warm-temperate low-to-high shrub/small tree | *Abutilon*, Amaranthaceae/Chenopodiaceae, *Arceuthobium*, Berberidaceae, *Caragana*, *Carpinus*, *Carpinus orientalis*, *Carpinus orientalis* type, *Carpinus orientalis*/*Ostrya*, *Celastrus*, *Cercis siliquastrum*, Chenopodiaceae, Cistaceae, *Cistus*, *Cistus incanus*, *Cistus salviifolius*, *Colutea*, Convolvulaceae, *Convolvulus*, *Cornus*, *Cornus mas*, *Cornus mas*/*Cornus suecica*, *Cotinus*, *Daphne*, *Elaeagnus*, *Erica*, *Erica* type, Ericaceae, Ericaceae type, *Euonymus*, *Fontanesia philliraeoides*, *Frangula*, *Frangula alnus*, *Fraxinus ornus*, *Genista* type, *Jasminum*, *Jasminum fruticans*, *Juniperus*, *Juniperus communis*, *Juniperus* type, *Lagonychium* type, *Lavatera* type, Leguminosae, *Ligustrum*, *Morus*, Myrtaceae, *Myrtus*, Oleaceae, *Paeonia*, *Paliurus*, *Paliurus spina-christi*/*Rhamnus*, *Paliurus*/*Rhamnus*, *Phillyrea*, *Phillyrea angustifolia*, *Pistacia*, *Prosopis*, Rhamnaceae, *Rhamnus*, *Rhamnus* subg. *Frangula*, *Rhododendron*, *Rhus*, *Rhus coriaria*, *Ruta*, Rutaceae, *Sambucus*, *Sambucus* type, Solanaceae, *Solanum*, *Syringa*, Thymelaeaceae, *Thymus*, *Ulex* type, *Vitex agnus-castus* |
| Xerophytic shrub | *Alhagi*, Amaranthaceae/Chenopodiaceae, *Artemisia*, *Artemisia* type, *Atraphaxis*, Capparidaceae, *Capparis*, Chenopodiaceae, *Chrozophora*, *Cistaceae*, *Cistus*, *Cistus ladanifer*, *Cistus salviifolius*, *Cotinus*, *Erica*, *Erica* type, Ericaceae, Ericaceae type, *Euphorbia*, *Juniperus*, *Juniperus* type, *Lycium*, *Myricaria*, *Nitraria*, *Ononis* type, *Paliurus*, *Paliurus spina-christi*/*Rhamnus*, *Paliurus*/*Rhamnus*, Rhamnaceae, *Rhamnus*, *Ruta*, Rutaceae, *Sarcopoterium*, Thymelaeaceae, *Thymus*, *Trachomitum*, *Zygophyllum* |
| Boreal cold-deciduous malacophyll broadleaved tree | *Alnus incana*, *Alnus viridis*, *Betula*, *Populus*, *Salix* |
| Boreal evergreen needle-leaved tree | *Abies*, *Picea*, *Picea abies*, Pinaceae, *Pinus*, *Pinus* (Haploxylon), *Pinus cembra*, *Pinus peuce* |
| Boreal needle-leaved deciduous tree | *Larix*, Pinaceae |
| Cool-temperate evergreen needle-leaved tree | *Picea*, *Picea orientalis*, Pinaceae |
| Eurythermic evergreen needle-leaved tree | Cupressaceae, *Cupressus*, *Juniperus*, *Juniperus communis*, *Juniperus* type, *Pinaceae*, *Pinus*, *Pinus* (Diploxylon), *Pinus* subg. *Pinus* |
| Temperate (frost-induced late budburst) cold-deciduous malacophyll broadleaved tree | *Acer*, *Acer platanoides*, Aceraceae, *Cornus*, *Cornus mas*, *Cornus mas*/*Cornus suecica*, *Corylus*, *Corylus avellana*, *Fraxinus*, *Fraxinus angustifolia*, *Fraxinus excelsior*, *Fraxinus excelsior* type, *Malus*, *Malus sylvestris* type, *Malus* type, *Populus*, *Prunus*, *Prunus spinosa* type, *Prunus* type, *Pyrus*, *Quercus*, *Quercus* (deciduous), *Quercus robur* type, *Salix*, *Sorbus*, *Sorbus* type, *Tilia* |
| Temperate (spring frost tolerant) cold-deciduous malacophyll broadleaved tree | *Acer campestre* type, *Aesculus*, *Carpinus*, *Carpinus betulus*, *Cercis siliquastrum*, *Fagus*, *Fagus sylvatica*, *Frangula*, *Frangula alnus*, *Fraxinus ornus*, Leguminosae, *Morus*, *Pistacia*, *Prunus* type, *Quercus*, *Quercus cerris*, *Quercus cerris* type, *Quercus frainetto*, *Quercus ithaburensis*, *Rhamnus* subg. *Frangula*, *Syringa*, *Ulmus*, *Ulmus glabra*, *Ulmus laevis*, *Ulmus*/*Zelkova* |
| Temperate (spring frost intolerant) cold-deciduous malacophyll broadleaved tree | *Carpinus*, *Carpinus orientalis* type, *Carpinus orientalis*/*Ostrya*, *Carya*, *Castanea*, *Castanea sativa*, *Celtis*, *Celtis reticulate*, *Ceratonia*, *Fagus*, *Fagus orientalis*, Juglandaceae, *Juglans*, *Juglans regia*, Leguminosae, *Liquidambar*, *Ostrya*, *Ostrya* type, *Parrotia* *persica*, *Platanus*, *Pterocarya*, *Pterocarya fraxinifolia*, *Punica*, Rhamnaceae, *Rhamnus*, *Styrax*, *Ulmus*/*Zelkova*, *Zelkova* |
| Temperate evergreen needle-leaved tree | *Abies*, *Abies nordmanniana*, *Cedrus*, Pinaceae, *Pinus*, *Pinus* (Diploxylon), *Pinus* (Haploxylon), *Pinus* subg. *Pinus*, *Pinus* *sylvestris*, *Taxus* |
| Warm-temperate evergreen malacophyll broadleaved tree | *Acacia*, *Acacia greggii*, *Acalypha*, *Citrus*, *Diospyros*, *Ficus carica*, *Ilex*, Leguminosae |
| Warm-temperate sclerophyll tree | *Acalypha*, *Arbutus*, *Buxus*, Leguminosae, *Nerium*, *Olea*, Oleaceae, *Quercus*, *Quercus* (evergreen), *Quercus calliprinos*, *Quercus coccifera*, *Quercus coccifera* type, *Quercus ilex*, *Quercus ilex* type, Rutaceae |
| Warm-temperate needle-leaved evergreen tree | Cupressaceae, *Cupressus*, *Juniperus*, *Juniperus sabina*, *Juniperus scopulorum*, *Juniperus* type, Pinaceae, *Pinus*, *Pinus* (Diploxylon), *Pinus* (Haploxylon), *Pinus pinaster* |
| Tuft tree | *Phoenix* |

Supplementary Table 7: Comparison of percent changes in biomes between adjacent samples for selected high-resolution Holocene pollen records using the new method and the standard biomisation approach. The biome with the lowest percentage of changes is highlighted in bold.

|  |  |  |  | **Biome change percentage** | |
| --- | --- | --- | --- | --- | --- |
| **Entity name** | **Lat - Lon** | **No. of samples** | **Age interval (cal. Years BP)** | **New approach** | **Standard biomisation** |
| Didachara core | 41.68 - 42.5 | 52 | 0 - 4071 | **8** | 45 |
| Lake Blatisto | 41.62 - 24.68 | 142 | 0 - 1626 | **15** | 43 |
| Kumisi core 1 | 41.58 - 44.83 | 30 | 0 - 1128 | **0** | 69 |
| Ispani II core 1 | 41.87 - 41.8 | 21 | 0 - 1862 | **0** | 10 |
| Imera core 1 | 41.65 - 44.22 | 34 | 0 - 2208 | **39** | 55 |
| Dead Sea DS7-1SC core | 31.49 - 35.44 | 55 | 0 - 2360 | **7** | 24 |
| Kumata Core | 42.59 - 23.25 | 19 | 0 - 1285 | 50 | **28** |
| Asi Gonia 2 | 35.25 - 24.28 | 41 | 0 - 1102 | **10** | 20 |
| Demiryurt Gölü Bottema Core | 39.73 - 37.38 | 21 | 0 - 1551 | 65 | 65 |
| Litochoro_core | 40.14 - 22.55 | 35 | 0 - 2234 | **24** | 29 |
| Aligol core 1 | 41.63 - 44.02 | 28 | 0 - 1360 | 26 | 26 |
| Iaz Core | 47.11 - 22.66 | 105 | 15 - 5315 | **13** | 30 |
| Capatana core | 46.47 - 23.14 | 60 | 16 - 2335 | 8 | **2** |
| Lake Almalou | 37.67 - 46.63 | 53 | 157 - 3402 | 27 | **15** |
| Melen Gölü | 40.77 - 31.05 | 76 | 165 - 4051 | **3** | 15 |
| Tsavkisi core 1 | 41.68 - 44.72 | 73 | 174 - 4575 | **31** | 33 |
| Amtkel 1 | 43.28 - 41.29 | 24 | 560 - 2002 | **17** | 26 |
| Sapanca long core SA03R6 | 40.72 - 30.26 | 31 | 776 - 1865 | **27** | 50 |
| Dry Lake 2 | 42.04 - 23.53 | 81 | 1295 - 6475 | **6** | 34 |
| Voulkaria | 38.87 - 20.83 | 51 | 1809 - 2947 | **0** | 18 |
| Maharlou Lake | 29.48 - 52.76 | 38 | 3625 - 5902 | 27 | **0** |
| Arkutino AR2 | 42.37 - 27.73 | 59 | 3702 - 7314 | **0** | 7 |
| Arkutino AR1 | 42.37 - 27.73 | 31 | 4705 - 6876 | **13** | 23 |
| Lake Varna (Beloslav-Poveljanovo) | 43.2 - 27.83 | 27 | 4986 - 6861 | 8 | **0** |
| Mohos1 | 46.08 - 25.92 | 92 | 5428 - 10991 | 23 | **9** |
| Giannitsa B | 40.67 - 22 | 28 | 7237 - 8674 | **41** | 59 |
| Steregoiu | 47.81 - 23.54 | 142 | 7273 - 12159 | 11 | **1** |
| GeoTü SL152 | 40.1 - 24.36 | 76 | 7531 - 10073 | **16** | 31 |
| Van - Wick Core | 38.53 - 42.47 | 137 | 8597 - 11705 | **1** | 13 |
| Taul Zanogutii core | 45.33 - 22.8 | 28 | 10556 - 11857 | **22** | 37 |

Supplementary Table 8: Optimal threshold for each biome used for detection of potential non-analogue assemblages.

| **Biome** | **Threshold** |
| --- | --- |
| CENF | 0.8245 |
| CMIX | 0.8812 |
| DESE | 0.8751 |
| ENWD | 0.8665 |
| GRAM | 0.8817 |
| TEDE | 0.8694 |
| TUND | 0.8835 |
| WTFS | 0.8450 |
| XSHB | 0.7987 |

**References**

Harrison, S. P. (2019). *Modern pollen data for climate reconstructions, version 1 (SMPDS)* [Data set]. University of Reading. https://doi.org/10.17864/1947.194

Hengl, T., Walsh, M. G., Sanderman, J., Wheeler, I., Harrison, S. P., & Prentice, I. C. (2018). Global mapping of potential natural vegetation: An assessment of machine learning algorithms for estimating land potential. *PeerJ*, *6*, e5457. https://doi.org/10.7717/peerj.5457

Marinova, E., Harrison, S. P., Bragg, F., Connor, S., Laet, V. de, Leroy, S. A. G., Mudie, P., Atanassova, J., Bozilova, E., Caner, H., Cordova, C., Djamali, M., Filipova-Marinova, M., Gerasimenko, N., Jahns, S., Kouli, K., Kotthoff, U., Kvavadze, E., Lazarova, M., … Tonkov, S. (2018). Pollen-derived biomes in the Eastern Mediterranean–Black Sea–Caspian-Corridor. *Journal of Biogeography*, *45*(2), 484–499. https://doi.org/10.1111/jbi.13128

Wei, D., Prentice, I. C., & Harrison, S. P. (2020). The climatic space of European pollen taxa. *Ecology*, *101*(8), e03055. https://doi.org/10.1002/ecy.3055
